# Comparative connectomics of *Drosophila* descending and ascending neurons

**DOI:** 10.1038/s41586-025-08925-z

**Published:** 2025-04-30

**Authors:** Tomke Stürner, Paul Brooks, Laia Serratosa Capdevila, Billy J. Morris, Alexandre Javier, Siqi Fang, Marina Gkantia, Sebastian Cachero, Isabella R. Beckett, Elizabeth C. Marin, Philipp Schlegel, Andrew S. Champion, Ilina Moitra, Alana Richards, Finja Klemm, Leonie Kugel, Shigehiro Namiki, Han S. J. Cheong, Julie Kovalyak, Emily Tenshaw, Ruchi Parekh, Jasper S. Phelps, Brandon Mark, Sven Dorkenwald, Alexander S. Bates, Arie Matsliah, Szi-chieh Yu, Claire E. McKellar, Amy Sterling, H. Sebastian Seung, Mala Murthy, John C. Tuthill, Wei-Chung Allen Lee, Gwyneth M. Card, Marta Costa, Gregory S. X. E. Jefferis, Katharina Eichler

**Affiliations:** 1 Neurobiology Division, MRC Laboratory of Molecular Biology, Cambridge, UK; 2 Drosophila Connectomics Group, Department of Zoology, University of Cambridge, Cambridge, UK; 3 Genetics Department, Leipzig University, Leipzig, Germany; 4 Research Center for Advanced Science and Technology, University of Tokyo, Tokyo, Japan; 5 Janelia Research Campus, Howard Hughes Medical Institute, Ashburn, VA USA; 6 Zuckerman Institute, Columbia University, New York, NY USA; 7 Department of Neurobiology, Harvard Medical School, Boston, MA USA; 8 Brain Mind Institute and Institute of Bioengineering, EPFL, Lausanne, Switzerland; 9 Department of Neurobiology and Biophysics, University of Washington, Seattle, WA USA; 10 Princeton Neuroscience Institute, Princeton University, Princeton, NJ USA; 11 Computer Science Department, Princeton University, Princeton, NJ USA; 12 Centre for Neural Circuits and Behaviour, University of Oxford, Oxford, UK; 13 FM Kirby Neurobiology Center, Boston Children’s Hospital, Boston, MA USA

**Keywords:** Neural circuits, Computational neuroscience

## Abstract

In most complex nervous systems there is a clear anatomical separation between the nerve cord, which contains most of the final motor outputs necessary for behaviour, and the brain. In insects, the neck connective is both a physical and an information bottleneck connecting the brain and the ventral nerve cord (an analogue of the spinal cord) and comprises diverse populations of descending neurons (DNs), ascending neurons (ANs) and sensory ascending neurons, which are crucial for sensorimotor signalling and control. Here, by integrating three separate electron microscopy (EM) datasets^1–4^, we provide a complete connectomic description of the ANs and DNs of the *Drosophila* female nervous system and compare them with neurons of the male nerve cord. Proofread neuronal reconstructions are matched across hemispheres, datasets and sexes. Crucially, we also match 51% of DN cell types to light-level data^5^ defining specific driver lines, as well as classifying all ascending populations. We use these results to reveal the anatomical and circuit logic of neck connective neurons. We observe connected chains of DNs and ANs spanning the neck, which may subserve motor sequences. We provide a complete description of sexually dimorphic DN and AN populations, with detailed analyses of selected circuits for reproductive behaviours, including male courtship^6^ (DNa12; also known as aSP22) and song production^7^ (AN neurons from hemilineage 08B) and female ovipositor extrusion^8^ (DNp13). Our work provides EM-level circuit analyses that span the entire central nervous system of an adult animal.

## Abstract (web summary)

Three electron microscopy datasets are combined to provide a complete connectomic description of the neural circuitry that makes up the neck connective in *Drosophila*, including the descending neurons, ascending neurons and sensory ascending neurons.

## Main

The nervous system’s exquisite control over body movement depends crucially on the bidirectional flow of motor and sensory information between the brain and the nerve cord. In insects, there are four principal classes of neurons that traverse the neck. The three most numerous are ascending neurons (ANs), which have their somata and dendrites in the ventral nerve cord (VNC); descending neurons (DNs), with somata and dendrites in the brain; and sensory ascending neurons (SAs), the somata of which reside outside the VNC. Finally, a small number of motor neurons (MNs) exit the neck connective before reaching the nerve cord, directly targeting neck muscles in the periphery^9^.

Light microscopy (LM) and genetic studies in *Drosophila* have shown that specific behaviours can be mapped onto individual neurons and circuits. LM images of genetic driver lines, mainly Gal4 drivers, are available in libraries such as Virtual Fly Brain^10^. These approaches have shown that a range of behaviours depend on individual DNs or on small groups of DNs, including^11^ aDN, DNg11 and DNg12 for anterior grooming sequences^12,13^; DNa02 for turning^14^; moonwalker DNs for backwards walking^15^; giant fibres for escape^16^; DNp07 and DNp10 for landing^17^; and DNp15, DNp20 and DNp22 for flight and neck control^18^. However, our understanding remains incomplete. Only a few studies have examined larger groups of DNs by morphology^5^ or behaviour^19,20^, and even less is known about ANs^21^.

Connectomics now offers the chance to reveal the detailed circuit mechanisms by which these neurons can exert powerful effects on behaviour. This starts with a complete enumeration and naming of the parts, and this alone can bring a change of scientific perspective. For example, in our first account of the complete cellular composition of the adult *Drosophila* neck connective in the male adult nerve cord (MANC) dataset, we found 1,328 DNs—almost twice the previous estimate of 700 DNs^1,22,23^. This dataset was an isolated nerve cord, but studying sensorimotor integration and control, and, specifically, the crucial role of DNs in exerting the influence of the brain on motor behaviour, requires us to understand the linked connectivity in the brain and nerve cord.

Male and female *Drosophila* exhibit sexually dimorphic behaviour, which is mediated by differences in both brain and VNC circuits. The sex of each *Drosophila* neuron is determined genetically, mainly through the expression of the transcription-factor genes *doublesex* (*dsx*) and *fruitless* (*fru*). Studies on *fru-* and *dsx-*expressing neurons and dimorphic behaviours have revealed several sexually dimorphic neurons and small circuits in the brain and VNC^24,25^. Females, for example, require oviDNs for egg laying^17,26^ and vpoDNs to open their vaginal plate when accepting a male^27^. By contrast, male-specific P1 central brain neurons control both intermale aggression and courtship steps such as wing extension^28,29^, and a set of DNs (pIP10 and pMP2) and at least six VNC cell types act to coordinate the time and shape of sine and/or pulse courtship song^7,29,30^. However, to understand how these neurons participate in complex, sexually dimorphic circuits, we must use methods that can reveal differences in connectivity, not just individual neuronal morphology, across the entire nervous system.

Herein, we describe all of the neck connective neurons of the female adult fly brain (FAFB-FlyWire)^2,3,31^ and the female adult nerve cord (FANC)^4,32^, and compare them with the MANC dataset^1,22,23^. We present strategies developed to bridge physically disconnected datasets (brain and VNC) and compare datasets of different sexes. Our work provides an integrative atlas of DNs, ANs and SAs based on EM connectome data from both the brain and the VNC. We then illustrate the utility of this complete and comprehensively annotated resource by addressing three scientific questions. First, we investigate the types of sensory information processed by DNs in the brain, and the connections between ANs and DNs in the brain and nerve cord. Second, we examine stereotypy across the three datasets at the level of morphology and connectivity. Finally, we define sexually dimorphic and sex-specific DNs and ANs and examine their circuits in the VNC. By studying complete ascending and descending pathways at synaptic resolution, we demonstrate a powerful method to understand motor control, which might inspire similar approaches in future studies of the vertebrate spinal cord.

## Matching neurons across three datasets

**Fig. 1:**
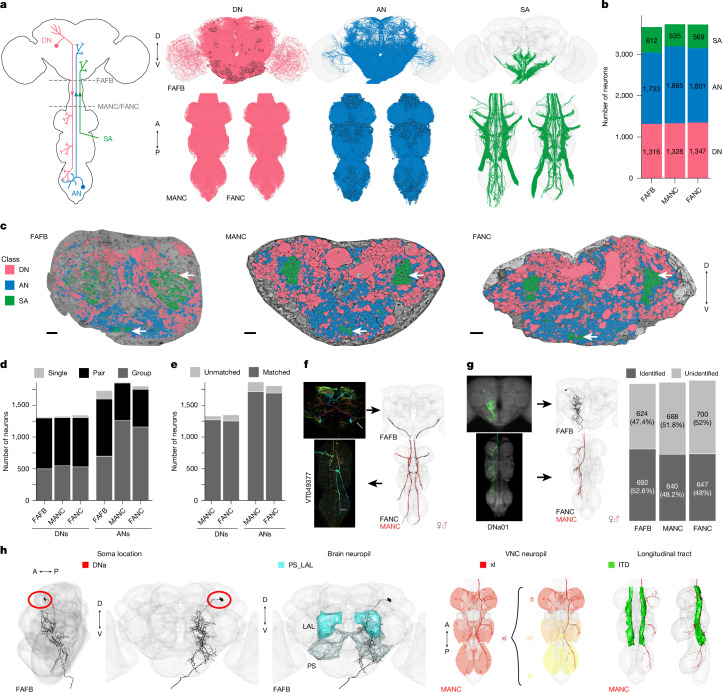
Reconstruction and identification of three neuronal classes across three datasets. **a**, Schematic of the CNS with the three neuronal classes that pass through the neck connective: DNs, ANs and SAs. FANC neurons are shown in MANC space here and in all following figures. A, anterior; D, dorsal; P, posterior; V, ventral. **b**, Number of neurons in each class and dataset. **c**, Transects through the neck of the three datasets: female adult fly brain (FAFB), male adult nerve cord (MANC) and female adult nerve cord (FANC). These neck connective transects were used as seedplanes to find and reconstruct the three classes of neurons shown in different colours. White arrows mark SA bundles. Scale bars, 5 μm. **d**, Number of DNs and ANs that have been left–right matched into pairs or groups in the three datasets. **e**, Number of DNs and ANs that have a match across the two VNC datasets. **f**, SAs were assigned modalities by matching to LM images. Left, example of a LM image of a femoral chordotonal organ club; white arrows point at the neuron of interest. Right, the EM reconstructions that were matched to the image. **g**, DNs were identified in all three EM datasets by matching the EM reconstructions to LM-level descriptions^5^ (see Supplementary File 4). Left, example of a LM image of DNa01 in the brain and VNC and next to it the FAFB, FANC and MANC EM reconstructions that were matched to those images. Right, quantification of DNs identified in all three datasets. **h**, DNs annotated by their soma location, brain and VNC neuropil innervation and the longitudinal tract they take in the VNC (DNa02 is used as an example). DNa, DNs with anterior dorsal soma; PS_LAL, posterior slope and lateral accessory lobe; xl, multiple leg neuropils; fl, front leg; ml, mid-leg; hl, hind leg; ITD, intermediate tract of dorsal cervical fasciculus. See Extended Data Figs. 3–6 for images of DNs across the neck connective, coloured by these four annotations.

**Extended Data Fig. 1:**
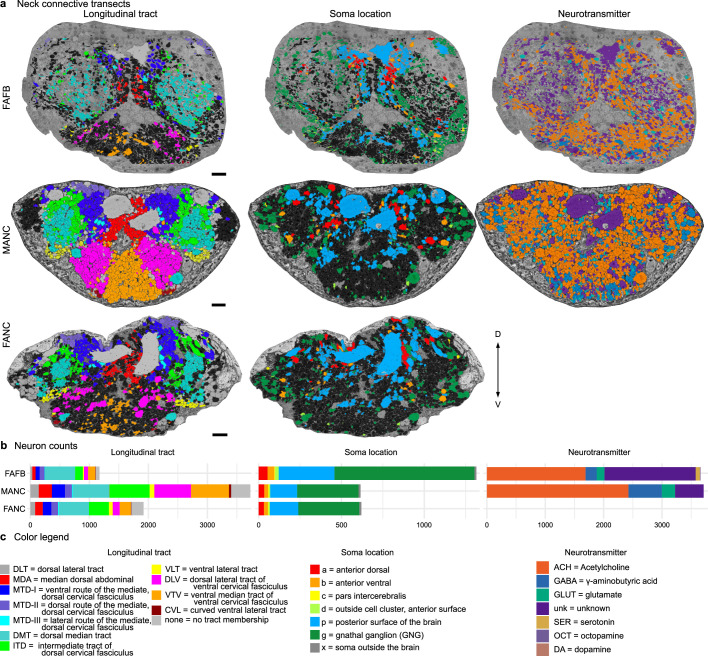
Cross section (frontal) of the neck connective in the three datasets. **a**, All neurons in the neck connective colour-coded by their longitudinal tract, soma location or predicted neurotransmitter^44^. **b**, Number of neurons by longitudinal tract, soma location or neurotransmitter in the three datasets. Neurotransmitter predictions are not yet available in FANC. **c**, Colour legend with details to the abbreviations used in **a**,**b**.

We reconstructed all of the neurons that traverse the neck connective in the female brain and nerve cord datasets (Fig. 1 and Supplementary Files 1 and 2), and compared these with the MANC^1,22,23^. Across the three datasets, we observe 1,315–1,347 DNs that transmit motor commands and other information from the brain to the VNC; 1,733–1,865 ANs that report processed sensory and motor state information from the VNC back to the brain; and 535–611 SAs that convey sensory information directly from the periphery to the brain (Fig. 1a,b). The position of these neurons in the neck connective is stereotyped, with DNs more dorsal, ANs more ventral and the SAs localized in two primary and two smaller bundles (Fig. 1c and Extended Data Fig. 1). DNs and ANs were matched across the two sides into pairs or groups in all datasets, and matched between the male and female VNC by their morphology and connectivity (Fig. 1d,e).

We have contributed our proofreading and annotation of these neck connective neurons to the separate online platforms hosting each of these EM datasets, such as the FlyWire connectome browser at https://codex.flywire.ai/. However, we have found that comparisons across datasets are more powerful when each dataset can be visualized simultaneously in the same virtual space with a common interface for querying and viewing annotations. We have therefore provided access to co-registered and uniformly annotated neck connective neurons. This combined three-dimensional web atlas can be viewed by following https://tinyurl.com/NeckConnective (see ‘Neuroglancer resource’ in the Methods for how this was made and https://github.com/flyconnectome/2023neckconnective for detailed instructions on how to use this interactive viewer as well as in depth programmatic analysis).

**Extended Data Fig. 2:**
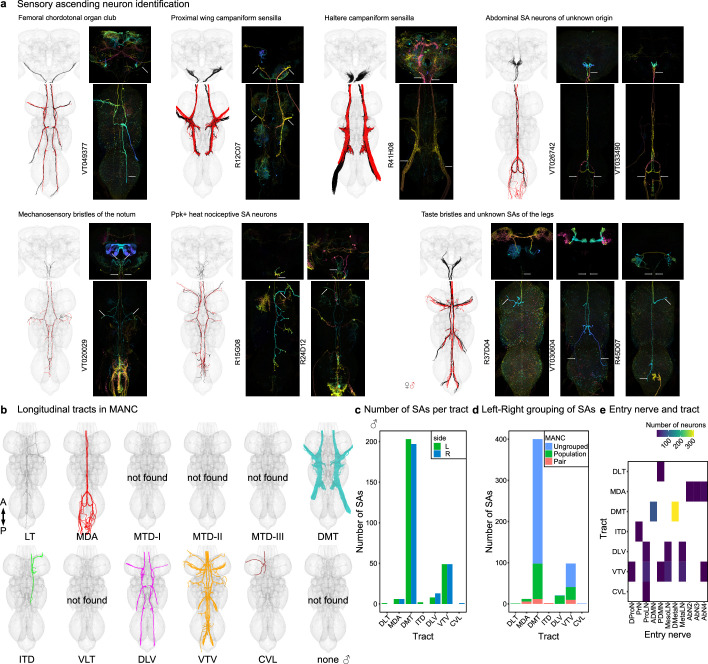
Sensory ascending neurons. **a**, Morphology of SAs identified in the three EM volumes. In black the EM morphology of DNs from female datasets (FAFB, FANC), in red from the male dataset (MANC). Next to them the LM images that allowed a grouping into sensory subclasses. **b**, Tract-based analysis of SAs in MANC. None of the SAs project along the MTD, or VLT tract. **c**, Number of SAs in each tract. **d**, Number of SA grouped into pairs or populations. **e**, Correlation of entry nerve to tract membership for MANC SAs^23^.

Currently available EM datasets comprise either the brain or the VNC and are therefore truncated at the neck during specimen preparation. This creates a considerable challenge for matching the brain and VNC parts of the neurons that send projections through the neck. Matching existing light-level descriptions of these neurons to their EM-reconstructed counterparts is necessary to identify these neurons across EM datasets, bridging the brain and the VNC, as well as linking morphology to behavioural data. The ANs and SAs have recently been typed in the male VNC (MANC) dataset^23^, but published LM information for these neurons is limited at present owing to a lack of driver lines. ANs will require detailed matching with future light-level resources, but we were able to make some specific matches (see Fig. 4). For SAs—a smaller and much less complex population—we were able to compare the EM-reconstructed neurons with available LM images of Gal4 lines from the FlyLight project^33^. We assigned gross sensory modalities for these neurons using the position of the tracts that they take through the brain and VNC (Fig. 1f and Extended Data Fig. 2; supporting evidence documented in Supplementary File 3).

**Extended Data Fig. 3:**
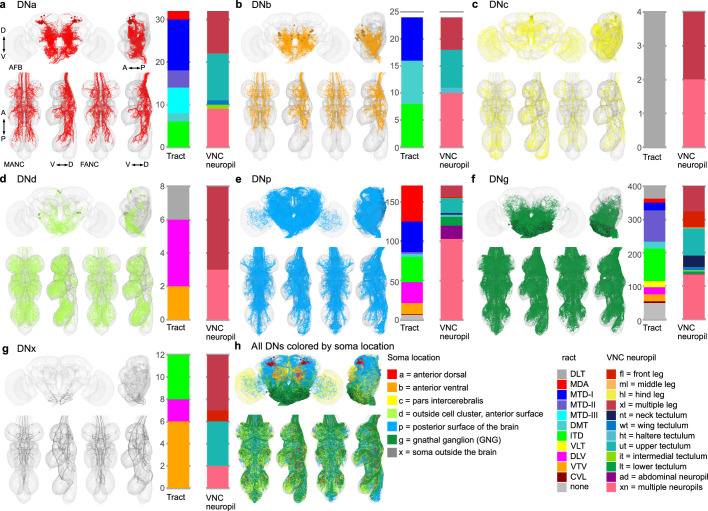
Morphology matched across the neck—soma location. Morphology of LM matched DNs across the three datasets colour-coded by cell body location according to a previous study^5^. **a**, DNa neurons have an anterior dorsal soma; **b**, DNb an anterior ventral soma; **c**, DNc a soma in the pars intercerebralis; **d**, DNd a soma in an anterior outside cell cluster; **e**, DNp are on the posterior surface; **f**, DNg are located in the GNG; **g**, DNx are outside the brain and **h**, all identified DNs coloured by soma location. In each panel the top images show reconstruction in FAFB in anterior and lateral view; the two bottom left images show MANC and two bottom right FANC in ventral and lateral view, respectively. The bar charts represent the distribution of the VNC longitudinal tract characteristics and VNC neuropil innervation for the neurons in each category - see colour legend.

**Extended Data Fig. 4:**
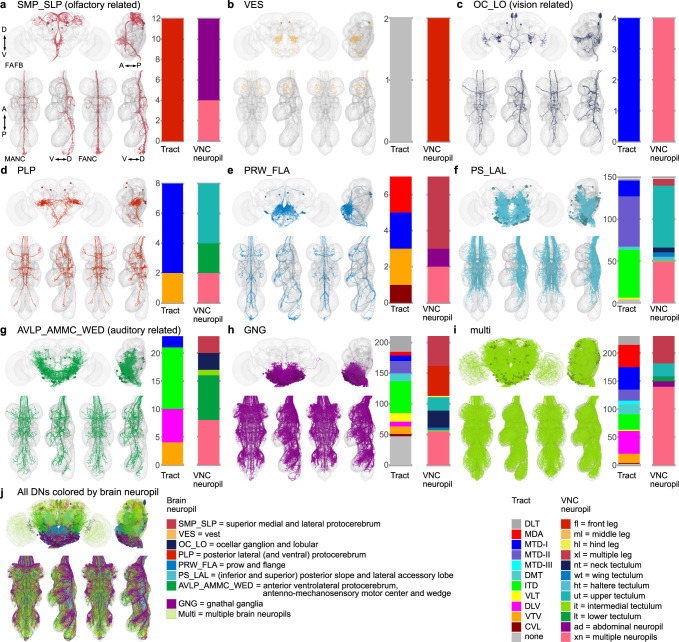
Morphology matched across the neck—brain neuropil. Morphology of LM matched DNs across the three datasets colour-coded by their brain neuropil innervation. DNs with input neuropil. **a**, Superior medial protocerebrum and superior lateral protocerebrum (SMP_SLP); **b**, vest (VES); **c**, ocellar ganglion and lobular (OC_LO, vision related); **d**, posterior lateral protocerebrum (PLP); **e**, prow and flange (PRW_FLA); **f**, posterior slope and lateral accessory lobe (IPS_SPS_LAL); **g**, antennal mechanosensory and motor centre and wedge (AMMC_WED, auditory related); **h**, gnathal ganglia (GNG); **i**, multiple innervations of neuropils across the brain (multi) and **j**, all identified DNs coloured by brain neuropil groups. In each panel the top images show reconstruction in FAFB in anterior and lateral view; the two bottom left images show MANC and two bottom right FANC in ventral and lateral view, respectively. The bar charts represent the distribution of the VNC characteristics longitudinal tract and VNC neuropil innervation for the neurons in each category - see colour legend.

**Extended Data Fig. 5:**
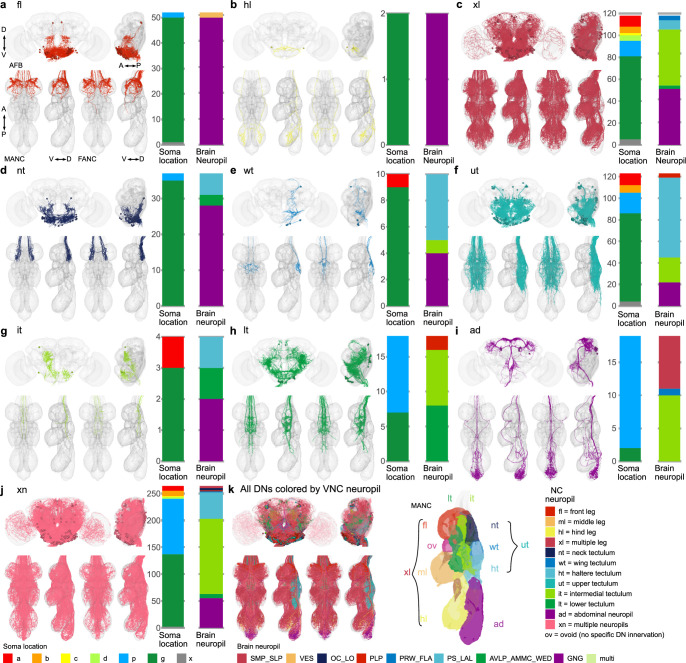
Morphology matched across the neck—VNC neuropil. Morphology of LM matched DNs across the three datasets colour-coded by VNC neuropil innervation. DNs with output neuropil. **a**, front leg (fl); **b**, hind leg (hl); **c**, multiple innervation in leg compartments (xl); **d**, neck tectulum (nt); **e**, wing tectulum (wt); **f**, multiple innervation into upper tectulum neuropils of the neck, wings and halteres (ut); **g**, intermediate tectulum (it); **h**, lower tectulum (lt); **i**, abdomen (ad); **j**, multiple innervations of neuropils across the VNC (xn) and **k**, all identified DNs coloured by VNC neuropil group^22^. In each panel the top images show reconstruction in FAFB in anterior and lateral view; the two bottom left images show MANC and two bottom right FANC in ventral and lateral view, respectively. The bar charts represent the distribution of the brain characteristics soma location and brain neuropil innervation for the neurons in each category - see colour legend.

**Extended Data Fig. 6:**
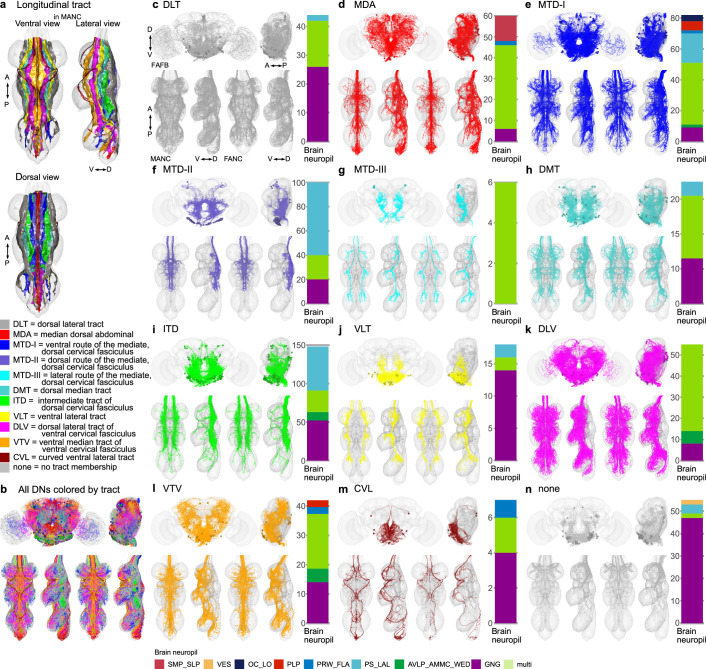
Morphology matched across the neck—tract. Morphology of LM matched DNs across the three datasets colour-coded by longitudinal tract membership in the VNC^22^. **a**, Overview of longitudinal tract in MANC as presented in another study^22^. **b**, All identified DNs coloured by the tract they take in the VNC. DNs in the tract. **c**, dorsal lateral tract (DLT); **d**, median dorsal abdominal tract (MDA); **e**, ventral route of the mediate tract of dorsal cervical fasciculus (MTD-I); **f**, dorsal route of the mediate tract of dorsal cervical fasciculus (MTD-II); **g**, lateral route of the mediate tract of dorsal cervical fasciculus (MTD-III); **h**, dorsal median tract (DMT); **i**, intermediate tract of dorsal cervical fasciculus (ITD); **j**, ventral lateral tract (VLT); **k**, dorsal lateral tract of ventral cervical fasciculus (DLV); **l**, ventral median tract of ventral cervical fasciculus (VTV); **m**, curved ventral lateral tract (CVL) and **n**, no tract membership (none). In each panel the top images show reconstruction in FAFB in anterior and lateral view; the two bottom left images show MANC and two bottom right FANC in ventral and lateral view, respectively. The bar charts represent the distribution of brain neuropil innervation for the neurons in each category - see colour legend.

In contrast to ANs and SAs, a substantial amount of LM image data exists from genetic driver lines for individual DNs. In parallel work, we have recently described all DN axons in the male VNC EM connectome (MANC)^22^ and matched some to previous LM images^5^. By overlaying EM morphologies on these LM images^5^ and a new LM collection^34^, we were able to identify 52.6% of FAFB and 48% of FANC DNs and increase the proportion of LM-identified DNs in MANC from 29% to 48.2% (Fig. 1g and Supplementary Files 4–7). DNs have previously been grouped by a range of characteristics, including their innervation of distinct neuropils—spatially localized regions dedicated to specific functions—in the brain and VNC. Here we show how the soma location, longitudinal main axon tract through the VNC and neuropil innervation compare across the three datasets^22^ (examples shown in Fig. 1h; details in Extended Data Figs. 3–6).

**Fig. 2:**
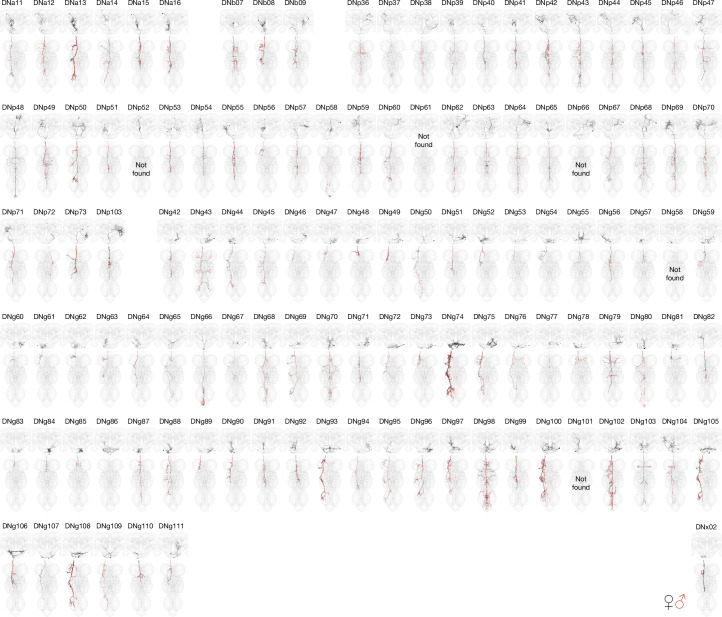
DN matching to new genetic driver lines. Morphology of identified DNs across all three datasets with nomenclature as described previously^34^. Four types could be found only in the brain (DNp52, DNp66, DNg58 and DNg101), and DNp61 is marked as not found because it is a duplicate. See Extended Data Fig. 7 for matching to DNs previously characterized at light level^5^. See Supplementary File 4 for details on the DN identification. DN morphologies from the female datasets (FAFB and FANC) are in black; the male dataset (MANC) is in red. DNs can be queried and viewed in interactive 3D at https://tinyurl.com/NeckConnective.

**Extended Data Fig. 7:**
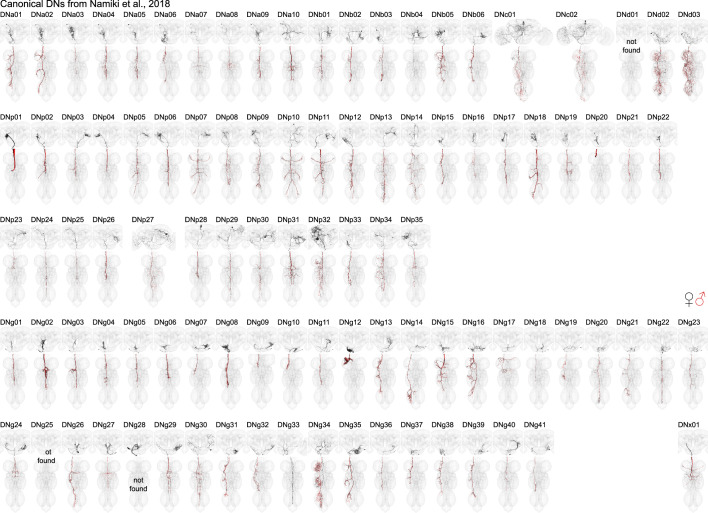
DN matching to genetic driver lines. Morphology of identified DNs across all three datasets with nomenclature as described in a previous study^5^. Two DN types could not be identified (DNd01, DNg25) in any of the three EM datasets and one DN type (DNg28) is only identifiable in the brain. See Supplementary File 4 for slide codes and for DN synonyms from the literature. In black the morphology of DNs from the female datasets (FAFB, FANC) in red from the male dataset (MANC). This figure is also provided in high resolution and DNs can be viewed in 3D at https://tinyurl.com/NeckConnective.

By separately matching the brain and VNC portions of a given DN to the same driver line, we were able to bridge connectome datasets. DNs have diverse morphologies in the brain and VNC that can be uniquely identified (Fig. 2; see ‘LM identification’ in the Methods). Of the 223 DN types identified by LM images from genetic driver lines, just 2 could not be found in any of our EM datasets; for 5 LM-identified types we could identify a matching type in the brain but were unsure in the VNC (Fig. 2, Extended Data Fig. 7 and Supplementary File 4). With this matching across datasets we could therefore analyse brain input and nerve cord output and compare this connectivity with a range of organizational features of the DN populations (Extended Data Figs. 3–6).

DN cell bodies are arranged in clusters, but as previously reported^5,22^, these soma locations do not correlate strongly with other organizational features. One exception is that DNa and DNb soma groups target leg and take-off or flight (upper tectulum) regions of the VNC, consistent with the known function for some of these neurons in steering during walking or flight (Extended Data Fig. 3a,b). Examining brain and VNC neuropil innervation patterns together highlights some notable correlations. DNs that innervate higher-order processing centres for olfactory stimuli (superior medial protocerebrum (SMP) and superior lateral protocerebrum (SLP) brain regions) mainly target the abdominal ganglion of the VNC, where they are likely to regulate reproductive or digestive functions (Extended Data Figs. 4a and 5i). DN axon tracts also assemble DNs into functional groups: DNs following the MTD-II tract consistently receive steering input in the brain (posterior slope and lateral accessory lobe regions) and target the upper tectulum in the VNC, which contains wing premotor circuits. Nevertheless, neuropil innervation and tract assignment remain coarse organizational features that provide only a guide to the sensory input or function of any given DN. We therefore performed a more detailed analysis of their connectivity.

## Sensory input onto DNs

**Fig. 3:**
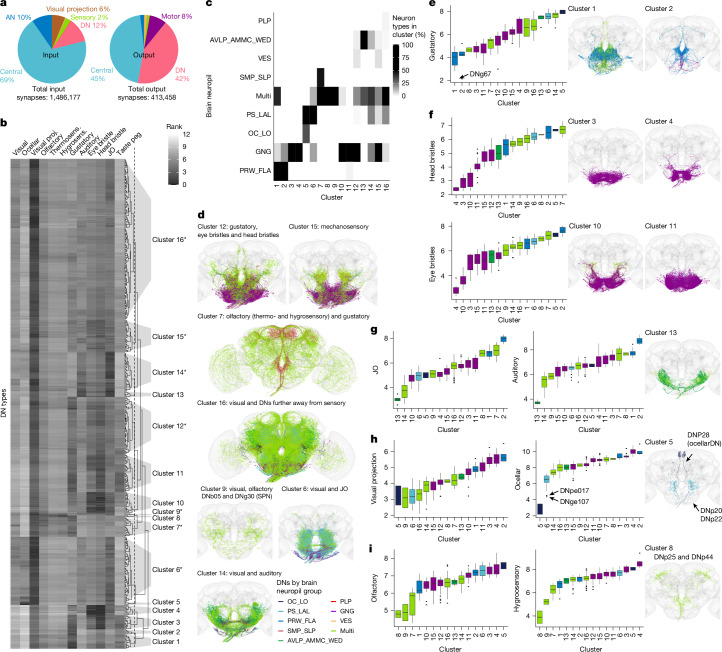
Sensory ranking of DNs. **a**, Pie charts showing the neuron class composition of input and output partners of all DNs in the brain (corresponds to FlyWire super_class). Total synapse numbers are shown at the bottom. **b**, Clustering of FAFB DNs by their sensory input rank (all apart from sensory DNs: DNx01, DNx02 and LN-DNs). The ranks, ranging from 1 to 12, taken from a previous study^2^, are defined as the traversal distances from a given sensory modality to each DN and then averaged by type. Low rank indicates a more direct connection from sensory modality to DN type. A cut height of 5 (dotted line in dendrogram) produces 16 clusters. JO, Johnston’s organ. Asterisks denote clusters with morphologies shown in **d**. **c**, Clusters shown in **b** by the brain neuropil assigned to DN types as a percentage of all DNs in that cluster (for abbreviations, see Extended Data Fig. 4). **d**, DN morphologies of clusters that are close in rank to several sensory modalities in the brain (clusters marked with asterisks in **b**). **e**–**i**, DN morphologies of clusters that are close in rank to one particular sensory modality (**e**, gustatory; **f**, mechanosensory: eye bristles and head bristles; **g**, mechanosensory: JO and auditory; **h**, visual projection and ocellar; **i**, olfactory: thermosensory and hygrosensory). Plots on the left show the average rank of the clusters defined in **b** for the different sensory modalities. The average rank is plotted for all 353 DN types in FAFB. Centre lines denote median; two hinges denote first and third quartiles; whiskers extend at most 1.5 × interquartile range (IQR) from hinge; and outlying points are shown. Arrows point to specific DN types that stand out. DN morphologies are plotted in their brain neuropil colours.

Our previous work provided a detailed description of how DNs connect to VNC motor circuits^22^. Now, by bridging the neck connective, we can define the sensory information received by all DNs in the brain (Fig. 3) and analyse how that relates to target circuits in the VNC. Summarizing DN input and outputs into six broad classes revealed patterns (Fig. 3a). Input is dominated by brain interneurons, conveying preprocessed sensory information (central and visual projection neuron classes; Fig. 3a). DNs also receive strong inputs (around 10%) from at least two other sources: ANs and DNs. AN inputs are likely to convey a mix of processed sensory and motor state information from the VNC; one long-standing hypothesis suggests that these connections are important for motor coordination^21^. DN–DN connections were more unexpected. We now find that DNs make a large number of output connections to other DNs in the brain (413,458 output synapses; Fig. 3a). This is noteworthy, because although many DNs make axon collaterals before leaving the brain^5^, their principal axonal arbours are considered to be in the VNC. DN connections with other DNs account for 42% of their total output in the brain but only 2% of their total output in the VNC^22^. This extensive DN–DN interconnectivity in the brain suggests the possibility of coordinated action across DNs, an idea that has recently been investigated by combining our connectome data with functional studies^35^. Many of these DN–DN connections are axo-axonic; whether this can result in direct excitation or inhibition of the downstream neuron or rather gates the axonal output of this neuron is unclear, although one team^35^ was able to show that optogenetic activation of some DNs can propagate to others. The remaining DN output in the brain mainly targets central brain interneurons (45%), including bilateral neurons that are likely to coordinate DN activity across brain hemispheres. Finally, DNs make 8% of their brain output directly onto MNs, mostly those that control the proboscis, the fly’s feeding organ.

**Extended Data Fig. 8:**
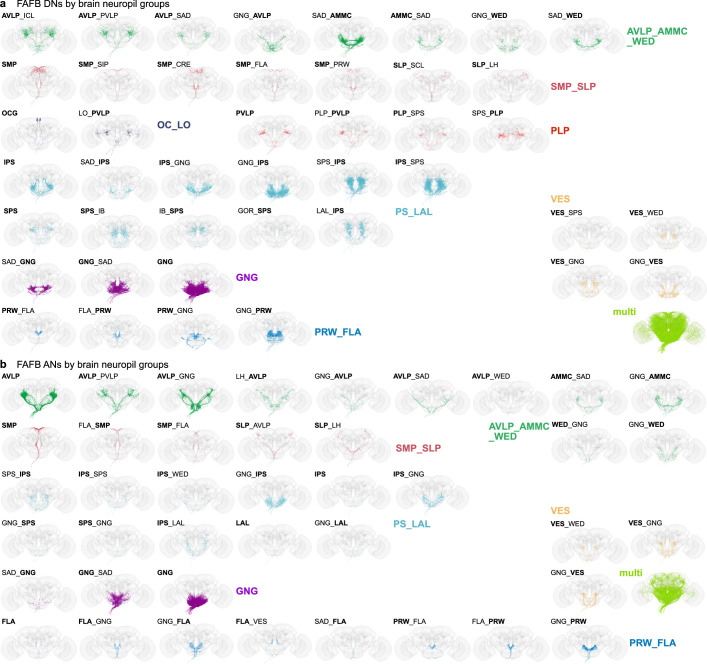
Brain neuropil groups. Morphology of DNs and ANs by primary brain neuropil. **a**, All DNs in FAFB-FlyWire were assigned one or two input neuropils. DNs that receive input from more than two neuropils are referred to as multi. **b**, All ANs in FAFB-FlyWire were assigned one or two output neuropils. ANs that output to more than two neuropils are referred to as multi. Morphologies are coloured by broader neuropil groups: auditory related neuropils (AVLP_AMMC_WED), higher-order multimodal sensory integration 1 (SMP_SLP), vision related (OC_LO), multimodal sensory integration 2 (PVLP, PLP), sensory modalities from the GNG (GNG, SAD), multimodal sensory integration and steering (PS_LAL), vest related (VES) and flange/prow (PRW_FLA).

DNs receive only a small fraction of their direct input from sensory neurons (2%). This is not particularly surprising, because direct sensory input to DNs bypasses higher processing regions of the brain. Therefore, understanding the sensory modalities that drive DNs requires more complex pathway analysis. Sensory neurons in the FAFB-FlyWire dataset have been extensively annotated, including their sensory modalities: visual, olfactory, gustatory, thermosensory, hygrosensory, auditory and mechanosensory neurons^3^. We examined the synaptic distance to DNs from these sensory modalities in the brain using a previously described information flow ranking^2^. This analysis excluded four types of DN that are themselves sensory neurons, referred to as sensory descending or SD (DNx01, DNx02, LN-DN1 and LN-DN2). DNs were assigned to 16 clusters on the basis of similarity in sensory input (Fig. 3b); these clusters typically have dendrites in the same brain regions even when the sensory information has already been preprocessed (Fig. 3c–i, Extended Data Fig. 8 for FAFB-FlyWire neuropil assignments and Supplementary File 5).

Nine small clusters are specific to individual sensory modalities (Fig. 3e–i). The first two DN clusters are the closest (lowest average rank) to gustatory sensory neurons and mainly arborize in two brain regions (prow and flange^36^) that receive taste information from the proboscis (Fig. 3b,c,e). Mechanosensory DNs fall into two groups, one (clusters 3, 4, 10 and 11) associated with eye and head bristles (Fig. 3f), and probably responsible for the highly targeted grooming of the corresponding bristle locations^37^, and another (clusters 13 and 14) close to antennal mechanosensory information and auditory cues^38,39^ (Fig. 3g).

Cluster 5 (Fig. 3h), is specific to visual inputs from the ocelli (rapid photosensors that signal head orientation relative to the sky); two of the three neurons, DNp20 and DNp22, are known to receive input from main optic lobe output neurons that encode pitch-associated or roll-associated optic flow and are involved in fast flight and neck motor control^18^.

There is only one small strongly olfactory cluster (8; Fig. 3i) containing two previously identified^40,41^ neurons, DNp25 and DNp44. The lack of large DN clusters associated selectively with vision or olfaction suggests that this sensory information is more likely to be integrated with other sensory modalities (for example, olfactory with gustatory; visual with auditory) or preprocessed in higher brain regions further away from DNs, as in cluster 16 (Fig. 3d).

Seven larger DN clusters integrate multiple sensory modalities (Fig. 3d, asterisks). By combining this sensory clustering with known behavioural roles for some neurons, we can make functional predictions across the whole DN population. For example, cluster-7 DNs integrate olfactory and gustatory information (Fig. 3d). This cluster includes the oviDN neurons, which integrate exactly this sensory information to select a nutrient-rich food source for egg laying^26,27^; we predict that other neurons in this cluster also control reproductive functions. Similarly, cluster-6 DNs receive a combination of visual and antennal mechanosensory information (sound and wind) within specialized brain neuropils (PS and LAL)^42^; these cues are essential for steering behaviours that have already been demonstrated^43^ for DNs in this cluster, such as DNb06. In summary, this analysis reveals the presence of specialized groups of DNs in which specific sensory inputs seem to be coordinated with distinct behavioural functions.

## DN and AN interactions

**Fig. 4:**
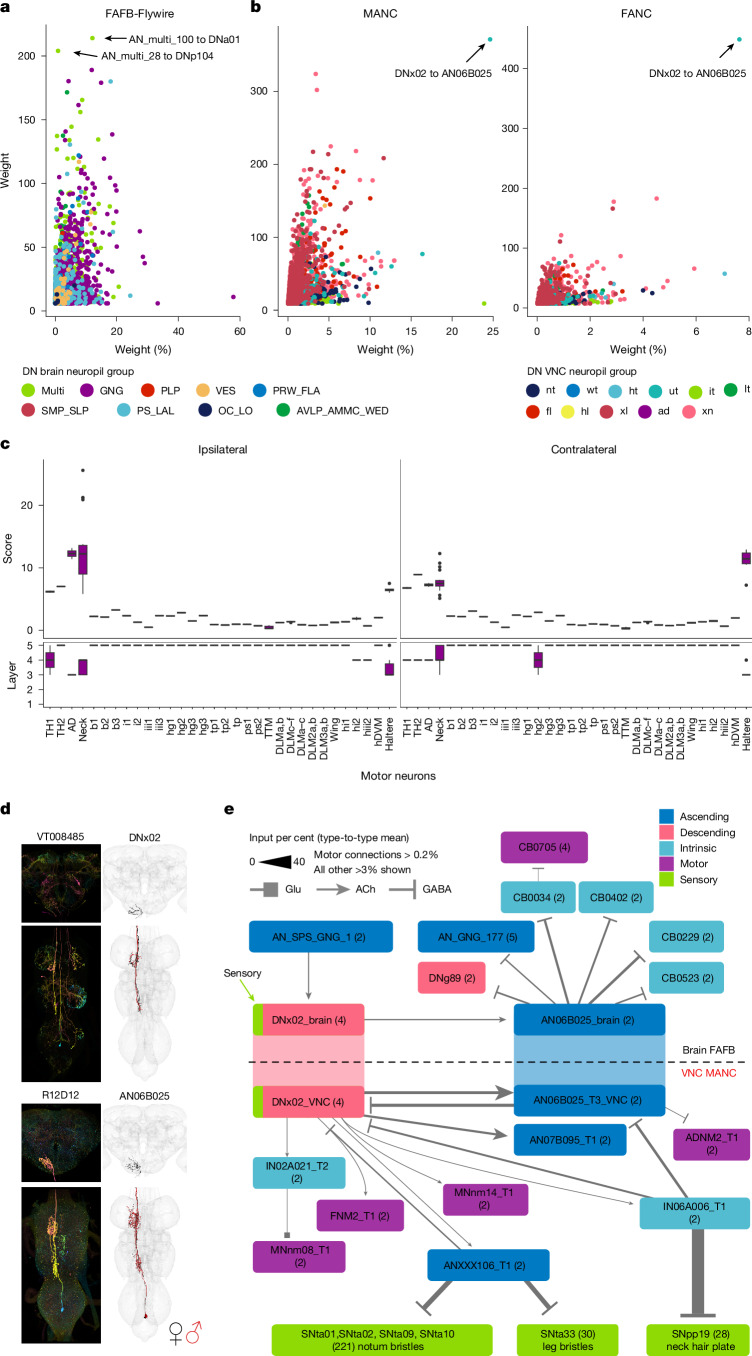
Direct DN and AN connections. Connectivity of ANs and SAs (ANs/SAs) to DNs and vice versa. **a**, Direct connectivity of ANs/SAs onto DNs in the brain. Connections between DNs and ANs/SAs are averaged by type and plotted by mean weight in per cent to mean weight. Arrows point to the two strongest connections in weight from ANs/SAs onto DNs. **b**, Direct connectivity of DNs in the VNC onto ANs/SAs. Connections are averaged by type, as in **a**. Arrows point to the one connection that stands out in both MANC and FANC. The weight in **a**,**b** is the number of presynapses. Dots are coloured by brain or VNC neuropil (see Extended Data Figs. 4 and 5 for abbreviations). **c**, Effective connectivity to MN targets ipsilateral and contralateral to the root side of DNx02 (*n* = 4). Centre lines denote median; two hinges denote first and third quartiles; whiskers extend at most 1.5 × interquartile range (IQR) from hinge; and outlying points are shown. **d**, Morphology of DNx02 and AN06B025 in the brain and VNC. The EM morphology from female datasets (FAFB and FANC) is in black; the male dataset (MANC) is in red. **e**, DNx02 circuit in the brain (FAFB-FlyWire) and in the VNC (MANC). Connections in both datasets are averaged by type and shown in the per cent input to the receiving neuron. AN_SPS_GNG_1 corresponds to AN06B057 in the VNC and targets the neck neuropil (see Supplementary File 12).

To guide specific sequences of behaviour in response to given stimuli, we expect feedback from VNC ANs back onto DN circuits in the brain (AN annotations and matching can be found in Supplementary Files 8–11). Strong direct connections between DNs and ANs are uncommon in the VNC and the brain (arrows point to connections with the highest weight in Fig. 4a,b; weight refers to number of synapses). One exception stands out in the VNC: the strong connection from DNx02 outputs onto AN06B025 (Fig. 4b,c). DNx02 are sensory DNs, two on each side, that enter the brain via the occipital nerve^37^ and have both direct and one-hop connections to neck MNs (Fig. 4c–e). Analogous to DNx01, which responds to mechanosensory stimuli on the antenna^5,20^, we predict that DNx02 responds to mechanosensory stimuli from the eye. We identified AN06B025 in the brain using a genetic driver line (Fig. 4d and Supplementary File 12), which enabled us to study the DNx02–AN06B025 circuit across the neck connective (Fig. 4e). Our analysis revealed that AN06B025 is also the top downstream target of DNx02 in the brain. AN06B025 is predicted to be GABAergic in both FAFB-FlyWire and MANC datasets^1,44^, suggesting that it inhibits DNx02. This defines a reciprocal AN–DN loop, a motif that was not observed in the *Drosophila* larval connectome^45^ (Fig. 4e).

On the basis of an analysis of the MN targets of this circuit (Fig. 4e, purple nodes), we propose that this loop coordinates head and leg movement during grooming and is triggered by the fly’s legs touching its eyes. In detail, the FNM2 MN should move the head upwards and inwards via the adductor muscle^9,22^; ADNM2 in the VNC and the cervical nerve MN (CB0705) in the brain both target the TH2 muscle, which promotes side-to-side movement of the head^9,46^. We suggest that DNx02 first moves the head upwards and inwards and is then inhibited by AN06B025, which also inhibits ADNM2 and disinhibits CB0705, potentially preparing for an extra sideways deflection as part of the head grooming sequence. Finally, through one hop in the VNC, DNx02 inhibits sensory neurons coming from leg and notum bristles and neck hair plate neurons, potentially dampening sensory information from these regions until the grooming movement is complete (Fig. 4e).

The DNx02–AN06B025 circuit is just one example of the kind of sensorimotor analysis made possible by our matching of brain and VNC neurons through the neck, across the entire CNS of *Drosophila*. The reciprocal loop circuit motif, in which X excites Y and Y inhibits X, can modulate the duration and magnitude of incoming excitatory signals and prevent an overly excited state, but can also lead to the emergence of oscillations, because the feedback inhibition of Y cannot fully suppress the firing of X^47,48^. It will be interesting to see whether this motif is common amongst other DN–AN combinations, including those required for sequential behaviours such as grooming^49^.

## Stereotypy in the VNC

**Extended Data Fig. 9:**
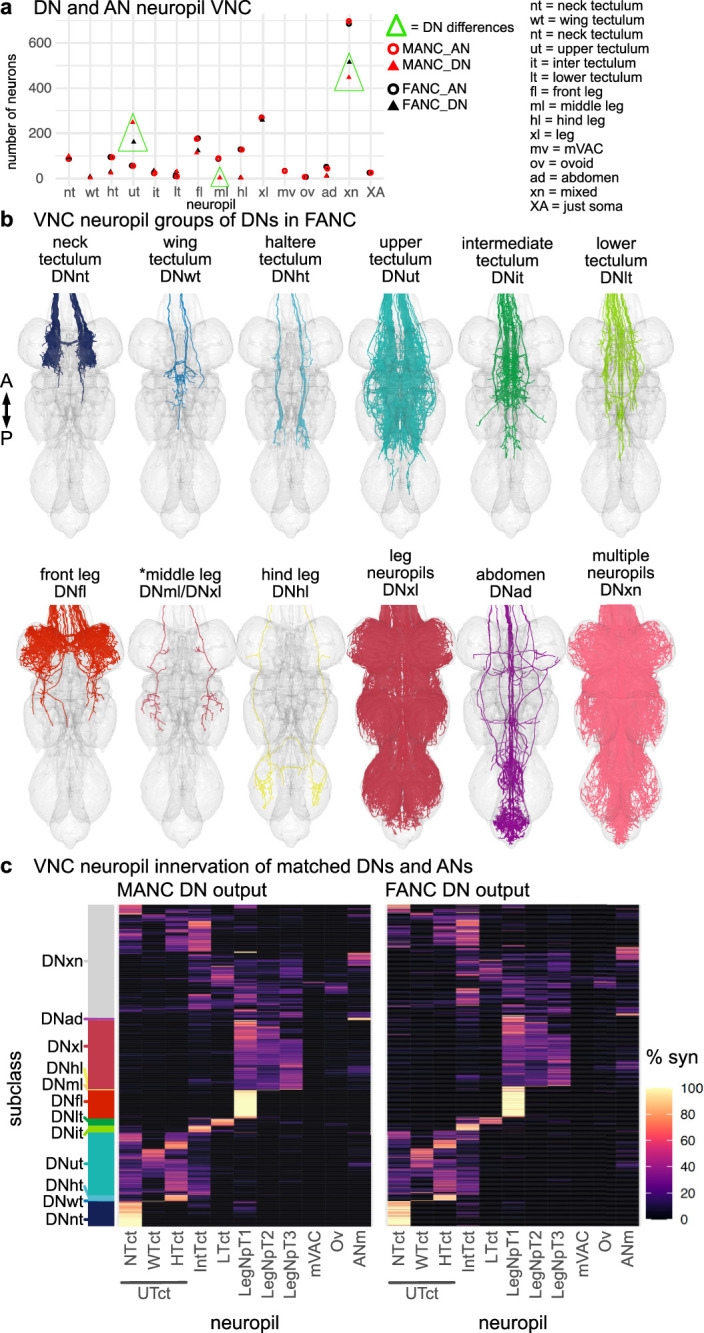
Neuropil-based analysis of DNs in FANC. **a**, Number of DNs/ANs assigned a VNC output/input neuropil in MANC and FANC. Green triangle indicates differences between datasets. A small number of DN types classified as upper tectulum DNs (DNut) in MANC fall below our 80% synaptic output threshold and thus are assigned to multiple neuropil innervating (xn). This is due to slight differences in neuropil meshes between the two datasets. **b**, Primary neuropil assignment of DNs in the FANC dataset to compare to previously published ones in the MANC dataset^22^. *the DN type targeting specifically the middle leg neuropil in MANC (DNml) has a considerable amount (>5%) of its synapses in the front leg neuropil in FANC, and is therefore in the neuropil category xl (for multiple leg neuropil innervating). **c**, Synaptic output in % by VNC neuropil of matched DNs in MANC and FANC. Each row represents one DN type, order is conserved between the two datasets. Left bar indicates the previously assigned neuropil-based subclasses from the MANC dataset^22^.

**Extended Data Fig. 10:**
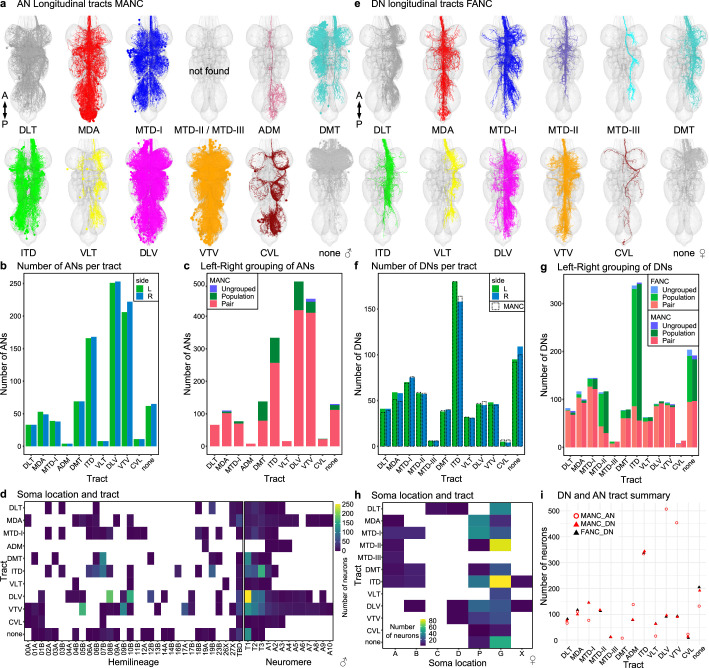
Tract-based analysis of DNs in FANC and ANs in MANC. **a**, Tract assignment of all left side DNs in the FANC dataset to compare to previously published tract assignment in the MANC dataset^22^. **b**, Number of DNs for each tract in comparison to MANC DNs (dotted line). **c**, DNs grouped into pairs or populations comparing FANC to MANC. **d**, Correlation of soma location and tract membership for identified FANC DN types based on LM images from genetic driver lines^5^. **e**, Tract assignment of all left side ANs in the MANC dataset. None of the ANs project along the MTD-II or MTD-III tract. A small additional tract was observed for ANs, referred to as AN-specific dorsal medial tract (ADM). **f**, Number of ANs in each tract. **g**, ANs grouped into pairs or populations comparing MANC to FANC. **h**, Correlation of hemilineage and neuromere to tract membership for MANC ANs. **i**, Number of DNs/ANs assigned a VNC long tract in MANC and FANC.

The datasets at hand offer an opportunity to compare neurons on both sides of the same individual fly, as well as across individuals and sexes, using two complete VNC datasets. These are interrelated issues, because we must understand normal variability when assessing whether differences between flies relate to a biological variable such as sex. Before analysing inter-dataset differences, we evaluated the stereotypy of neuronal morphology and connectivity. DN and AN populations were matched to LM-described types (97% of LM-defined types found in all three datasets; Fig. 2 and Supplementary File 4) across sides and datasets (92–99% neurons matched Fig. 1d,e), and we quantified their consistency in tract and VNC neuropil innervation (Extended Data Figs. 9 and 10), revealing a high degree of stereotypy (matching in Supplementary Files 5–11).

**Fig. 5:**
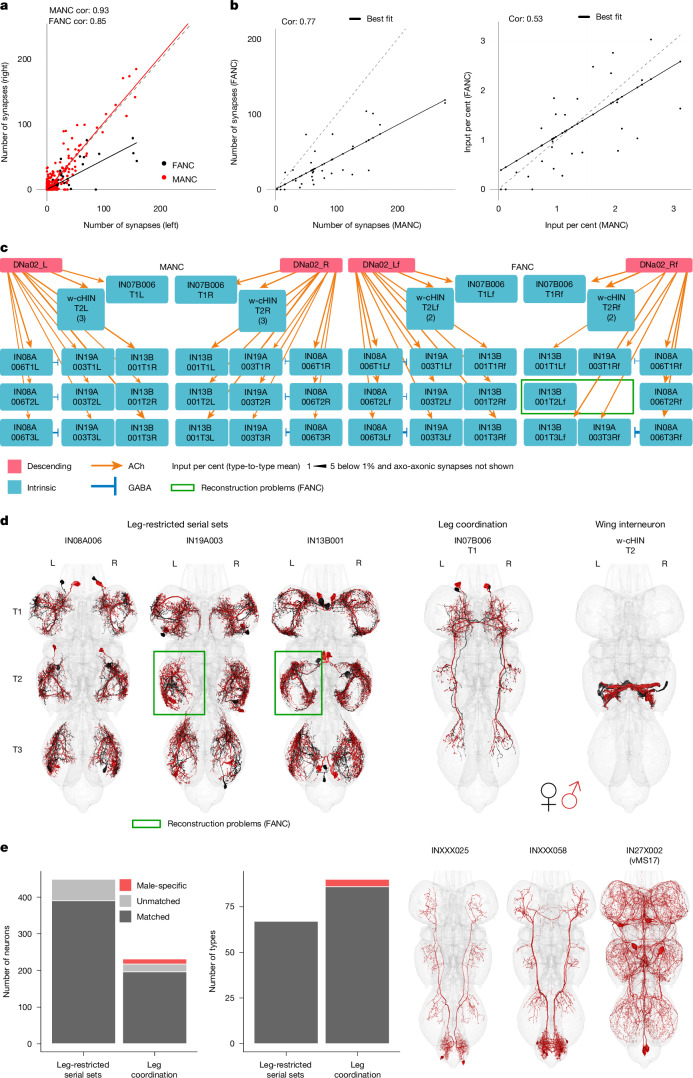
Comparisons across VNC datasets. **a**–**d**, DNa02 as an example of a stereotyped circuit in the VNC. This analysis is provided at https://github.com/flyconnectome/2023neckconnective/blob/main/code/3_DNa02_downstream.Rmd. **a**, DNa02 output partners in MANC and FANC compared across sides of the same dataset. Best-fit slope is 1.02 for MANC and 0.45 for FANC. **b**, DNa02 output partner types that receive more than 1% of DNa02 output (all but three matched between MANC and FANC). Left, raw synapse numbers (best-fit slope = 0.42); right, the same neurons shown by the input per cent (best-fit slope = 0.69) to the receiving neuron. The Pearson correlation coefficient (Cor) is shown for **a**,**b**. **c**, Top partner types targeted by DNa02 in MANC and FANC: three sets of serial leg-restricted neurons (IN08A006, IN19A003 and IN13B001), the w-cHIN and a bilaterally projecting neuron (IN07B006). Arrow thickness corresponds to the per cent input to the receiving neuron, and only values higher than 1% are shown. All nodes are single neurons apart from the w-cHIN, number in brackets. **d**, EM morphologies of the neurons shown in the connectivity graphs in **c**. Reconstructions from FANC are in black; those from MANC are in red. **e**, MANC leg premotor circuit neurons (by number of neurons and type) published in a previous study^22^, matched to FANC. All leg-restricted serial sets were found, although some are missing on one or the other side in FANC. All apart from three types of leg coordination neurons were matched to FANC. EM morphologies of those three unmatched types are shown on the right as potentially male-specific neurons.

Having assessed the degree of stereotypy in cell numbers and cell types, we moved on to connectivity between identified cell types. We decided to use DNa02 as an example: it has a well-defined function in producing turns during walking, a behaviour common to both sexes^14,43^. We and others^43^ comprehensively reconstructed DNa02 downstream partners in the partially proofread FANC dataset, enabling robust comparisons with the completed MANC dataset^22^. We matched downstream partners across hemispheres (Fig. 5a) and datasets (Fig. 5b). Focusing on strong partners (more than 1% of DNa02 output), we could see that these were highly consistent populations: three serially repeated local neuron sets each controlling one leg; a bilaterally projecting neuron coordinating across legs; and the w-cHIN neurons, which control wing MNs. All cell types were cross-identified, with just one partner missing on one side of one segment in FANC owing to reconstruction issues (Fig. 5c), and morphologies were also highly consistent (Fig. 5d). We also compared connection weights in the fly connectome as defined by the number of unitary synapses between neurons; these were highly correlated across hemispheres (Fig. 5a) albeit with a somewhat lower score in FANC. When comparing datasets, we saw that DNa02 downstream connections in FANC were significantly weaker (best-fit slope = 0.42). We therefore also computed a normalized connection score (percentage of input onto each downstream partner), and saw that these have a slope closer to unity (0.69). In short, these neurons form consistent circuits across connectome datasets; differences in absolute (but not relative) connection weights are likely to be of technical origin owing to lower synapse recovery in FANC (see ‘Connectivity’ in the Methods).

These results gave us a detailed picture downstream of a single pair of DNs. We next sought to extend our observations to larger networks of neurons. We focused on premotor circuits of the leg, which we have described in MANC as containing 67 neuron types local to a single leg (448 neurons) and 75 leg-interconnecting types (231 neurons). Premotor neurons of at least the front legs have been extensively reconstructed in FANC^50^, and their serially repeated nature (6 or 12 neurons per type; that is, 1 or 2 per leg) ensure identification even when reconstruction status is more uneven.

We successfully identified matches to all 67 local neuron cell types by morphology and connectivity clustering (Fig. 5e and Supplementary File 13). For interconnecting neurons, 3 out of 75 types were missing in FANC: INXXX025, INXXX058 and IN27X002 (Fig. 5e; morphologies on the right). These might be male-specific or so sexually dimorphic in morphology that we cannot confidently match them. The first two types (INXXX025, which is predicted to be cholinergic, and INXXX058, which is predicted to be GABAergic) project from the abdominal ganglion to the leg neuropils, and would be good candidates for male-specific leg movements; for example, in response to abdominal curling during copulation. In support of this, IN27X002 matches vMS17 neurons that have been previously reported to control male courtship song^7^.

On the basis of an initial automated segmentation^4^, at the time of writing the FANC community has proofread just over 5,000 neurons (including the 1,804 ANs reported in this study)^23^. Although complete matching of all 23,500 VNC neurons across the female and male datasets is still in progress, the 4,517 neurons we have matched so far (including DNs, ANs, SAs and the leg premotor circuit; Supplementary File 2) exhibit highly stereotyped morphology and connectivity across both sides, and across repeated VNC segments and datasets. Before this work, there had been little matching of precise cell types between the FANC and MANC datasets, with the notable exception of foreleg and wing MNs, and until now, the FANC^4,50^ and MANC^22^ datasets had only been analysed independently.

## Sexual dimorphism

**Fig. 6:**
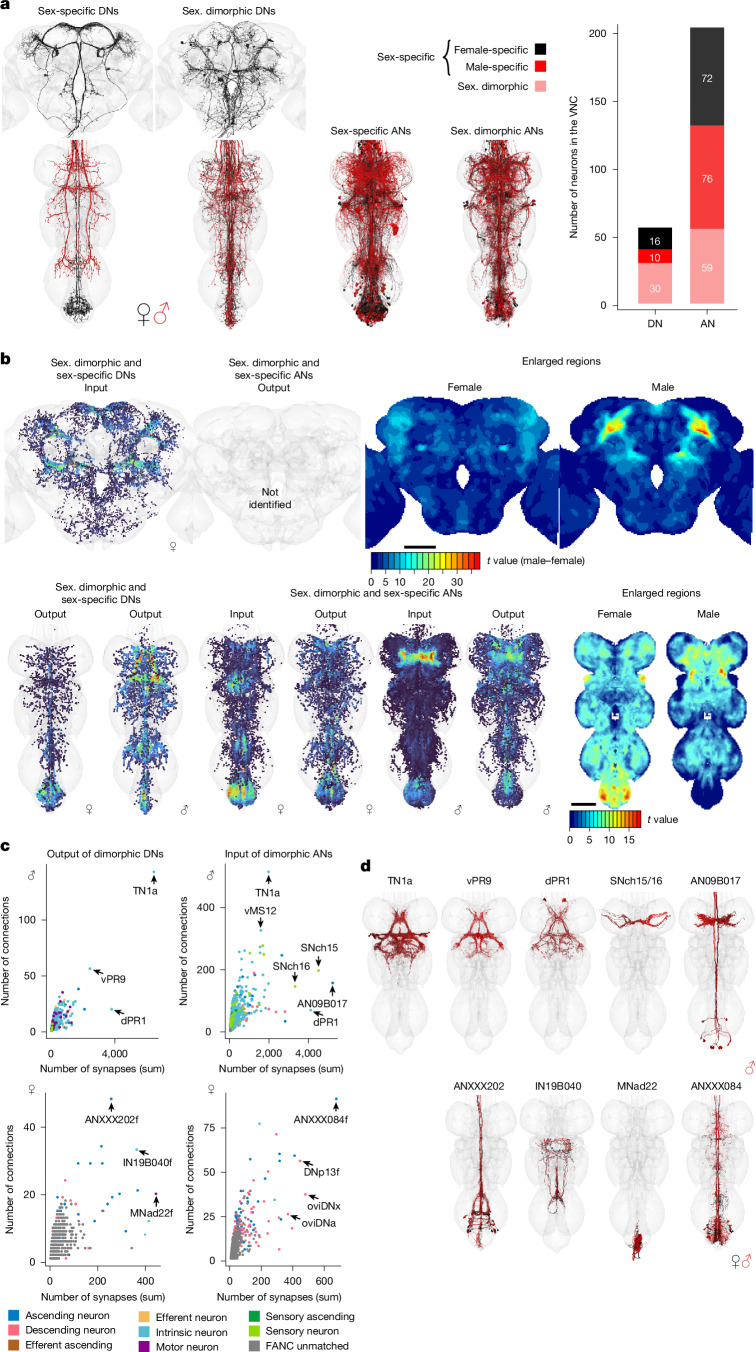
Sex-specific or sexually dimorphic neurons. **a**, Morphology of DNs in three datasets and ANs in the two VNC datasets that are sexually dimorphic (sex. dimorphic) or sexually specific (sex-specific) as described in the literature or predicted by the matching. The EM morphology from female datasets (FAFB and FANC) is in black; the male dataset (MANC) is in red. The number of sexually dimorphic and sex-specific DNs and ANs in the VNC can be found on the right. **b**, Density of pre- or postsynapses of the DNs and ANs shown in **a** compared with previously published images^52^ of enlarged regions in the female and male CNS. **c**, Downstream or upstream partners of sexually dimorphic or sex-specific DNs or ANs in FANC and MANC. Arrows point to the strongest partners by number of synaptic connections and number of neurons connecting onto them. **d**, Reconstructions of partner neurons in MANC (**c**, top row) or in FANC that were matched to MANC neurons of that type.

EM datasets are scarce, but this study provides an opportunity to compare across sexes using two VNC datasets. One of the main challenges in this process is that the absence of a neuron in one dataset might have several explanations: these include differences in connectome reconstruction, inter-individual biological variation or actual sexual dimorphism. Our work on neck connective neurons (and many of their partners) now defines a complete subset of the connectome that has been fully reconstructed and annotated across all three datasets. With this groundwork in place, the next step was to establish clear criteria for what qualifies as dimorphic or sex-specific in connectome analysis. We defined potentially female- or male-specific (sex-specific) DNs and ANs as neurons that are well reconstructed and can be confidently paired across the two sides of one VNC, but cannot be matched across the VNC datasets. We consider neurons to be potentially sexually dimorphic (sex. dimorphic in Fig. 6) if they differ in morphology between the two VNC datasets, but are morphologically consistent across both sides of each nervous system.

We first identified the set of unmatched neurons between the VNC datasets: 59 MANC DNs versus 97 FANC DNs and 155 MANC ANs versus 115 FANC ANs (Fig. 1e). After detailed analysis, we concluded that 60% of this pool are most likely to be sex-specific or sexually dimorphic; the remaining 40% are probably examples of L–R biological variation or are affected by segmentation issues. This 60% included all 22 (11 types) sexually dimorphic or sex-specific DNs that have previously been reported^6,8,26,27^ (see Supplementary Files 14 and 15 for details), as well as 34 new DNs (17 types); for ANs, only 20 out of 205 neurons (5 of the 69 types) were previously reported. We first performed some aggregate analyses of these populations.

On the basis of our definitions, 1% of all DNs (16 female and 10 male neurons) and 4% of all ANs (70 female and 76 male neurons) are sex-specific, whereas 2% of DNs (30 neurons) and 3% of ANs (59 neurons) are sexually dimorphic in morphology (Fig. 6a). We then visualized brain and VNC regions in which sex-specific and sexually dimorphic neurons receive and send information (Fig. 6b). In the female FAFB-FlyWire brain, we observed that inputs to dimorphic DNs are concentrated in the ring of the protocerebral complex^51^, suggesting that male enlarged regions can be used to identify zones of both male- and female-specific connectivity^52^ (Fig. 6b). Dimorphic DN output in the VNC also aligns with sex-enlarged structures: the abdominal ganglion (enlarged in females) is the site of both male- and female-specific synapses, whereas the male-enlarged triangular region associated with the song system^52^ (Fig. 6b, right) receives male-specific DN input. Turning to dimorphic ANs, there is a concentration of female-specific input in the abdominal region. Conversely, in males, the input to dimorphic ANs is concentrated in the front leg (T1) sensory area, the site of male-specific contact pheromone receptors.

Sexually dimorphic and sex-specific DNs and ANs represent only a small fraction of the total neuron population, and it is unclear how widespread an effect they have. One simple answer to this question is that although less than 3% of DNs have sex differences, these connect with 12% of DN partners (1,596 out of 12,964 neurons with 10 or more synapses). Examining the top downstream neurons (Fig. 6c, left), we see a few very strong connections in males to the song circuit: TN1a (silencing decreases sine song), vPR9 (silencing alters the amount of pulse and sine song) and dPR1 (silencing increases sine song)^7^. The top input partners of dimorphic ANs (Fig. 6c, right) include the song circuit neurons already mentioned, as well as two sets of sensory neurons coming from foreleg (T1) taste bristles (SNch15 and SNch16, both midline-crossing)^23,53^; dimorphic ANs such as AN09B017 and AN05B035 also extensively interconnect this T1 leg sensory area. AN09B017 seems to match *fru*-expressing vAB3 neurons, which transmit contact pheromone signals from the front legs to P1 neurons in the brain^54^.

**Extended Data Fig. 11:**
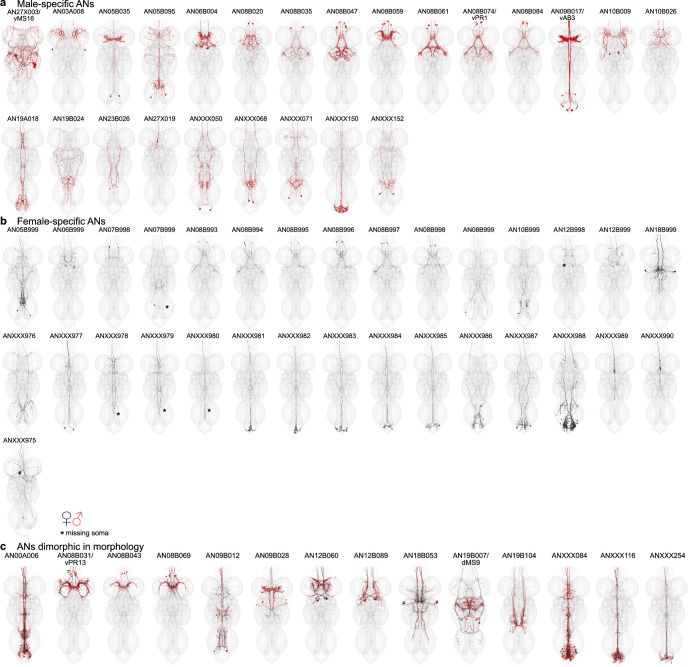
Potentially sexually dimorphic or sex-specific ANs in the VNC. **a**, Morphology of all the potentially male-specific ANs by type. **b**, Morphology of the potentially female-specific ANs by newly assigned types. **c**, Morphology of the potentially sexually dimorphic ANs by MANC type names. In black the EM morphology from the female dataset (FANC) in red from the male dataset (MANC). Stars indicate ANs with missing soma in FANC due to missing EM image data.

In FANC, the top output partners of dimorphic DNs also seem to be dimorphic themselves. ANXXX202 has dimorphic dendritic arbours in the abdominal ganglion; IN19B040 has differences in morphology; abdominal MNad22, which we could not link to any previous studies, is not obviously dimorphic in morphology but receives multiple sex-specific inputs. Input partners to dimorphic ANs include three notable dimorphic DNs: oviDNa, oviDNx and DNp13, as well as the dimorphic ANXXX084 (see Fig. 6d for morphologies). Thus, signals from these DNs that control ovipositor (egg-laying) circuits are rapidly fed back to the brain via dimorphic ANs (ANXXX981–ANXXX983 and ANXXX988; Extended Data Fig. 11); however, these ANs also receive local sensory inputs on their VNC dendrites, which might be indicative of actual execution of motor commands.

In summary, in the VNC male dimorphic DNs and ANs are heavily connected with male-specific song circuits and sensory information from the front legs, whereas female dimorphic DNs and ANs are particularly focused on abdominal reproductive circuits. We next show how we can use these combined connectome resources to study specific circuits in detail.

## Dimorphic DNs in mating and egg laying

**Fig. 7:**
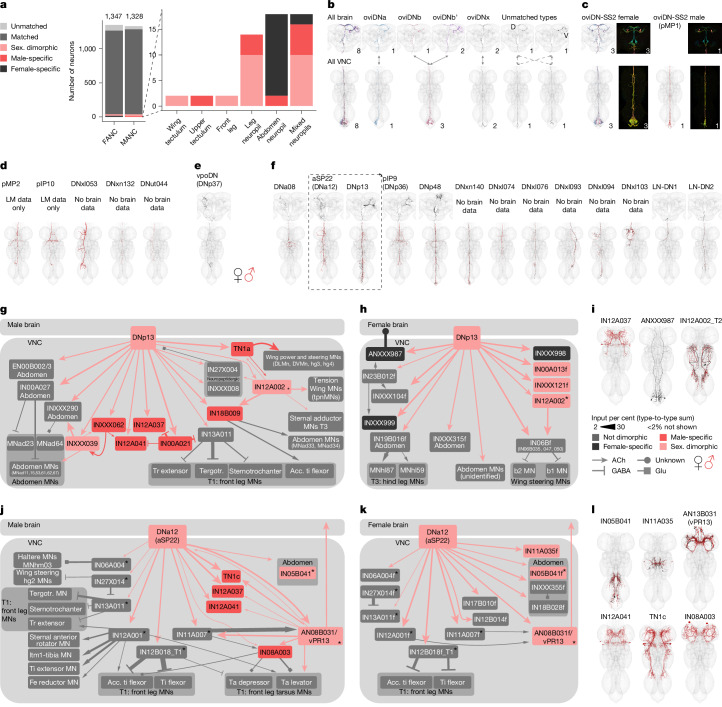
Sexually dimorphic and sex-specific DNs. **a**, Proportion of DNs that are sex-specific or sexually dimorphic by dataset and primary input neuropil. **b**, Morphology of DNs in the three datasets belonging to the oviDN hemilineage. Two of the six types are uniquely identifiable but we cannot match them across the neck (unmatched types, oviDNd and oviDNv). **c**, EM morphologies identified within the LM images from a female and a male of the oviDN-SS2 line. **d**, EM morphologies of previously LM-characterized male-specific DNs and new potentially male-specific DNs. **e**, EM morphology of the female-specific DN vpoDN (DNp37). **f**, EM morphologies of LM-characterized sexually dimorphic DNs and new potentially sexually dimorphic DNs. **g**,**h**, Connectivity downstream of the sexually dimorphic DNp13 in MANC (**g**) and FANC (**h**). There is just one downstream partner in common between the two sexes, IN12A002 (marked with an asterisk). All other partners are sex-specific (coloured black or red) or dimorphic in their connections (pink), or exist in both datasets but are not downstream of DNp13 in the other dataset (coloured grey). The female-specific INXXX998 is also a downstream target of vpoDN. Tergotr., Tergotrochanter; Acc. ti flexor, accessory tibia flexor. **i**, EM morphology of some of the top VNC targets. **j**,**k**, Connectivity downstream of the sexually dimorphic DNa12 (aSP22) in MANC (**j**) and FANC (**k**). There are eight downstream neurons in common. Only T1 leg MNs have been systematically identified between the two datasets, so other FANC leg MNs are not shown. Asterisks indicate common downstream partners of dimorphic DN pairs. Tr, Trochanter; Ti, Tibia; Fe, Femur; Ta, Tarsus. **l**, EM morphology of some of the top VNC targets. The EM morphology from female datasets (FAFB and FANC) is in black; the male dataset (MANC) is in red.

DNs frequently act as powerful command neurons; each sex-specific or dimorphic type will be an entry point to circuits of biological interest, so we now briefly catalogue them (Fig. 7a). The oviposition-promoting oviDNs are the best known female-specific DNs. These closely related neurons share a common cell body fibre tract and were previously reported to consist of six neurons divided into two subtypes based on genetic driver lines^26^. Combining these data with total EM reconstruction of the oviDN tract, we have now defined six female oviDN types (16 neurons) and one male type, pMP1, which is similar to female oviDNx (Fig. 7b,c). We identified five male-specific DN types in the VNC. pMP2 and pIP10 are known to control male song^29,55^; the three newly identified neurons target other VNC domains, including leg circuits (Fig. 7d). In addition to the oviDNs, we identified one female-specific DN, vpoDN (DNp37), which has previously been described as important in female receptivity by controlling opening of the vaginal plate^27^ (Fig. 7e). We also identified 14 dimorphic DN types present in both sexes; LM-level matches for 7 types (DNa08, aSP22, DNp13, pIP9, DNp48, LH-DN1 and LH-DN2), further support this finding and allowed identification across both VNC datasets and the female brain (Fig. 7f and Supplementary File 14). Each DN has complex downstream circuits. We selected two dimorphic cell types for detailed study across both sexes: DNp13 and DNa12 (aSP22), which both regulate male and female mating behaviours^6,8,56^.

DNp13 is highly dimorphic in morphology and connectivity, but the male and female neurons are still clearly more similar than any other cell type in each connectome (Fig. 7f). In both sexes, their ultimate targets are wing and abdominal MNs (Fig. 7g,h). However, different MNs are targeted through different VNC interneurons in each sex, consistent with different behavioural outputs. DNp13 (also known as pMN1^57,58^) in females responds to courtship song and its activation drives ovipositor extrusion; this tube-like organ is used for egg laying but is proposed to be a sexual rejection signal in this context^8^. Unsurprisingly, abdominal MNs are top targets of DNp13 in FANC (Fig. 7h). However, we also see that DNp13 targets three interneuron types (denoted IN06Bf in Fig. 7h) that output strongly to b1 and b2 wing MNs; we note that wing flicking is another female rejection behaviour^59^, albeit not one that has been linked to DNp13.

Turning to MANC, we see a strong DNp13 connection to the TN1a interneuron, which promotes sine song^55^. We also find that there are several strongly connected wing MNs directly downstream (Fig. 7g). At present, there are no behavioural reports for DNp13 in males, but this suggests a potential role in song production or perhaps in agonistic wing interactions between rival males^60^. The only common downstream target of DNp13 in males and females is IN12A002 (2% threshold; Fig. 7g,h, black star, morphology shown in Fig. 7i). IN12A002 has similar morphology in both sexes, but different connectivity. The remaining intrinsic neuron targets (grey in Fig. 7g–i), are present in both datasets but only connect to DNp13 in one sex.

DNa12 (aSP22) is not as dimorphic as DNp13 (Fig. 7f,j,k). Activation of DNa12 elicits proboscis extension, spontaneous posture adjustments, front leg extension and abdomen movements in both sexes^6^. However, the type of abdominal movement elicited by DNa12 differs between males (abdominal bending, as in copulation) and females (abdominal extension, perhaps related to oviposition movements). Consistent with this, we see that DNa12 neurons share 8 out of 12 MANC and 8 out of 13 FANC downstream partners (threshold of >2% input). We strengthened this observation by matching all 38 downstream partners of MANC DNa12 in FANC: only the male-specific types (TN1c, IN12A037, IN12A041 and IN08A003) were missing.

DNa12 connections to the front leg tibia extensor MNs can be found in both MANC and FANC, and activation of DNa12 promotes foreleg lifting in both sexes^6^; this should allow males to clasp females during copulation, but the function in females is not clear. DNa12 connects to the sexually dimorphic AN08B031 in both sexes, but AN08B031 has distinct downstream partners: dimorphic cell types can therefore still make monomorphic connections (Fig. 7j–l). We were unable to identify LM images of genetic driver lines for this AN, but still hypothesize that it could coordinate proboscis extension and foreleg lifting in males, both late-stage courtship actions. Although DNa12 has no reported song phenotype, we observe a strong connection to the TN1c pulse song neuron^55^ (both direct and through dimorphic AN13B031; Fig. 7j,k). Finally, we observe two noteworthy downstream partners of DNa12 in FANC: IN05B041 and INXXX335. These target the abdominal ganglion and might be responsible for the dimorphic abdomen extension in females (Fig. 7k).

These two detailed examples show how we can combine genetic driver lines and the published literature with male and female connectomes to obtain mechanistic insights into sex-specific behaviour. We were able to link previously studied neurons into circuits, identify previously unstudied neurons with key circuit locations and form new behavioural hypotheses about the function of known neurons; for example, roles in song or wing movement for DNp13 and DNa12.

## Sex-specific ANs of the 08B hemilineage

The results in the previous section considerably expand our understanding of sexually dimorphic DNs. However, for ANs, the existing knowledge base is much lower. Previous work has shown that ANs signal information about behavioural states^21^ (such as forward or backward walking, turning and grooming) but only a handful of genetically identifiable ANs have been reported^15,61–63^. The situation for sexually dimorphic ANs is even sparser: the vast majority (64 out of 69 types) are, to our knowledge, reported here for the first time (morphologies shown in Extended Data Fig. 11).

**Fig. 8:**
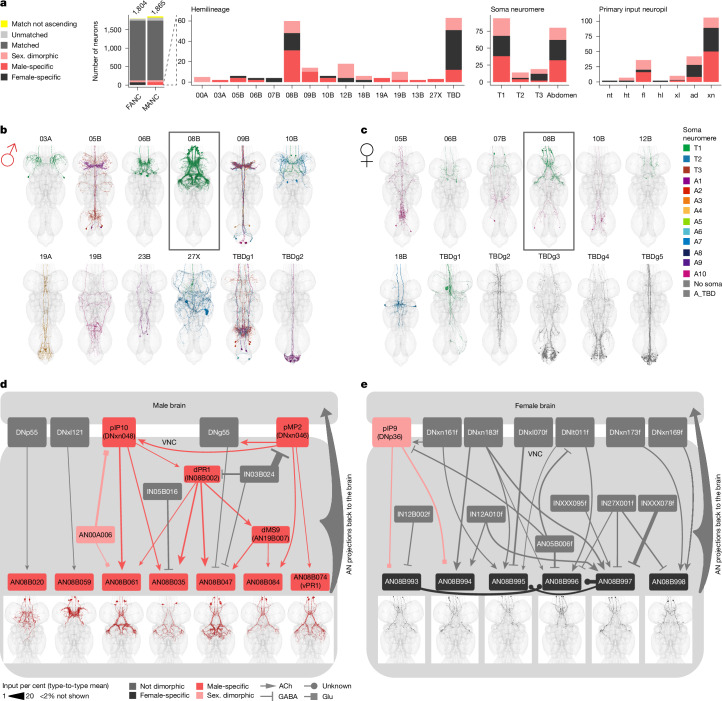
Sexually dimorphic and sex-specific ANs. **a**, Proportion of ANs that are potentially sex-specific or potentially sexually dimorphic by hemilineage, soma neuromere and primary input neuropil. **b**,**c**, Morphology of ANs that are potentially sex-specific in males (**b**, MANC neurons) and females (**c**, FANC neurons) by hemilineage. FANC neurons in **c** were assigned hemilineages and soma neuromere if possible and given new type names. **d**,**e**, Input circuit in the VNC to potentially sex-specific AN types of hemilineage 08B with soma location in T1 (black box in **b**,**c**). **d**, Male-specific ANs. **e**, Female-specific ANs. AN08B074 is the previously described male-specific AN vPR1^51^, which as previously hypothesized receives input from DNxn046 (pMP2). Morphology of AN types underneath. All input neurons with more than 2% input onto the receiving AN are shown. FANC neurons in **e** were matched to MANC neuron types by morphology and connectivity and given the MANC names with an addition of f for female.

We confirmed the soma location for all dimorphic ANs in FANC, and compared neuron number across the two datasets (Fig. 8a and Supplementary File 15). Neurons in the adult nervous system are generated by genetically defined neuroblasts (stem cells), which make packets of cells called hemilineages with tightly bundled cell body fibres at stereotyped locations in the CNS^3,23^. We found that hemilineage 08B contains the largest number of dimorphic ANs. There is also a substantial number of dimorphic ANs in the abdominal ganglion (associated with reproduction functions), but this is unfortunately partially missing in FANC. We therefore focused on the 08B hemilineage, which contains both male- and female-specific ANs; these are the only sex-specific ANs that clearly innervate the mesothoracic triangle, a strongly dimorphic VNC area that contains many neurons crucial for male song production^7,51,52^ (Fig. 8b,c).

There are seven types of male-specific 08B AN (Fig. 8d). Five of these (including the one previously identified type, vPR1; ref. ^51^) form an interconnected circuit with known song neurons such as the dimorphic DNs pMP2 and pIP10, intrinsic neuron dPR1 and sexually dimorphic AN19B007 (dMS9). The last two types (AN08B020 and AN08B059) also have arbours in more ventral neuropils associated with the legs and locomotion rather than song, and are therefore likely to be involved in different male behaviours (Fig. 8d).

We also defined six types of female-specific 08B AN (Fig. 8e). They share no upstream partners with the seven male-specific types, suggesting considerable divergence in function. Consistent with this, one type receives input from the sexually dimorphic *fru*-expressing pIP9 (DNp36)^51^. Although the other upstream partners are all sex-shared, they do not connect to any 08B ANs in males, confirming that there is major rewiring at this circuit node.

We propose that male-specific 08B ANs provide feedback to the brain during male song production, perhaps acting as a corollary discharge^64,65^ to suppress auditory responses to self-generated song. The female-specific 08B ANs might transmit sexually dimorphic information (such as sexual receptivity and post-mating state) back to the brain, a pathway which either takes a different course or does not exist in the male nervous system. Conceptually, these examples showcase two distinct forms of dimorphic circuit logic: (1) sex-specific neurons can interact with one another to produce a sex-specific behaviour; and (2) circuit elements present in both sexes interact with sex-specific neurons, thereby forming a neuronal ensemble that carries sex-specific information.

## Discussion

Through detailed reconstruction and annotation across three EM connectome datasets, here we present a complete set of neuronal classes spanning the neck connective. We have categorized the neurons by sensory modality (for DNs and SAs in FAFB-FlyWire) and neuropil innervation across the brain and nerve cord, as well as tract and soma location. We have established a platform for systematic neuron typing based on light-level and cross-dataset identification. Early access to the proofread and annotated connectome data that we describe has already enabled and assisted a range of work, including studies of the circuit basis of locomotor behaviour and the organizational and functional logic of DNs^35,43,50,65–68^.

This connectomic platform allows us to infer the circuit basis of behaviours, including sexually dimorphic patterns, and formulate hypotheses about numerous circuit components. Future studies of any part of the CNS can now link genetically defined neurons, physiological responses or optogenetic manipulations to the connectome. Whereas earlier studies often focused on a single neuropil of the brain (for example, the antennal lobe for odour processing or the mushroom body for learning and memory), connectomics has already underlined that the sensorimotor circuits underlying behaviours are complex and brain-spanning. A recent connectome-constrained neural network of the motion pathways in the optic lobe of *Drosophila* reliably predicted measurements of neural activity from the connectivity of 64 cell types^69^; this approach relied on optimizing the neural output of the modelled system to solve a behavioural task. Our work should enable the modelling of key output neurons of the brain, and also models spanning the entire CNS.

Despite the common challenge of small sample sizes in connectomics, our study stands out in that it uses multiple datasets across individuals and sexes; previous work compared specific (isomorphic) circuits in *Drosophila* larva^70,71^, and recent work in adults has compared two female brain connectomes^3^. By systematically comparing DNs, ANs and SAs from both male (MANC) and female (FANC) VNC datasets, we categorized similarities and differences between the two sexes. This represents the first—to our knowledge—comprehensive comparison of *Drosophila* neuronal morphology and connectivity between sexes at EM resolution. We have identified all previously published dimorphic DNs and describe the circuits of DNa12 and DNp13 in both datasets. Moreover, we excluded and annotated differences that seem to be biological variation between individuals (including rare cases of missing neurons) or reconstruction state in one dataset. We enumerate potential sex-specific or sexually dimorphic DNs and ANs, with a specific focus on circuits of sex-specific ANs from the 08B hemilineage, which is associated with male song during courtship. This lays the groundwork for understanding circuits for sex-related behaviours all the way from the sensory periphery, through higher brain processing to motor output.

Acquiring large EM volumes remains extremely challenging, and each dataset had some quality issues. For example, there is a localized truncation of part of the dorsal abdominal ganglion in the FANC dataset. There are also a few cases in which cell bodies are missing from the surface of the VNC; in such cases, we could not at first be certain whether a particular cell was an AN or a SA, but this could be resolved by comparison across datasets. Neurotransmitter predictions, although available in two of the three datasets, lack neuropeptide predictions and receptor expression data, an important gap given that neurotransmitters such as glutamate can be excitatory or inhibitory. Matching cell types bilaterally within datasets and especially across datasets still requires time and skill (even when augmented by LM images from genetic driver lines). However, by making deductions using the full range of annotations, leveraging bilateral consistency within each dataset and using powerful across-dataset connectivity clustering strategies, almost all cases could be resolved. This underscores the importance of rich and comprehensive connectome annotation to guarantee their quality and comparability. Nevertheless, the lack of comprehensive light-level descriptions and genetic driver line libraries for ANs remains a challenge in linking brain and VNC connectomes. Forthcoming *Drosophila* full CNS datasets^72^ will address these gaps, building on the foundations established in this study. Indeed, the Neuroglancer scenes with our neurons from this work are already transformed into the virtual CNS space of the male CNS dataset^72^.

In the future, new datasets, including larger mammalian connectomes, will be acquired from different specimens, in different conditions and by different research groups. Identifying and matching types across EM datasets, taking into account biological variation and technical issues such as neuronal truncation, will be an essential part of using these datasets. Our work in *Drosophila* provides strategies to extract notable differences from just two or three datasets, including rigorous definitions for sexual dimorphism that will enable future comparisons of connectomes. Finally, establishing general principles of sensorimotor coordination from synaptic-resolution wiring diagrams in the fly will benefit researchers studying locomotor and premotor circuits across species. Whether insect and vertebrate nerve cords evolved independently or share a common ancestor, as suggested by the concept of dorsoventral axis inversion^73^, examining similarities and differences in spinal cord and VNC organization should provide valuable insights.

## Methods

### Neck connective proofreading and annotation

We defined a perpendicular plane through the neck connective posterior to the cervical nerve for the two VNC datasets (MANC and FANC), and anterior to the cervical nerve for the FAFB dataset. Supplementary Files 1 and 2 include the FANC_seed_plane and FAFB_seed_plane, which list all profiles with their *xyz* coordinates in this plane, IDs and the neuronal class. Every neuronal profile passing through these planes in FANC or FAFB was individually reviewed, reconstructed and annotated by manual proofreading of the corresponding automated segmentations. We reviewed 3,874 profiles (which received a total of 117,450 edits) in FANC, and 3,693 profiles (which received 131,207 edits) in FAFB. Both datasets provide open community-based proofreading platforms (see https://flywire.ai/ and https://github.com/htem/FANC_auto_recon/wiki), and some of these edits were due to general proofreading in each volume, but most were from our comprehensive proofreading of neck connective neurons. The first pass review of the MANC neck connective was performed in mid-2021; for FAFB the first review periods were late 2020–early 2021 and again in mid-2022. After the initial review of ANs and SAs in the VNC datasets, neurons were assigned a putative soma side programmatically, directly or indirectly through a MANC mirroring registration^22^. Neurons were mirrored based on their soma side or their neck plane side and NBLAST clustered^74^. This analysis allowed for an initial grouping of left–right homologous sets and enabled the identification of neurons with different morphologies on each side of the nervous system, triggering further proofreading (because these differences usually resulted from residual segmentation errors). The combination of comprehensive proofreading of the whole dataset followed by within-dataset matching and focused proofreading was essential to ensure high-quality connectome data and annotation. Most DNs and ANs have a unique morphology and were grouped into pairs; otherwise, neurons were combined into larger groups containing more than one neuron per side. This was the case especially for SA neurons in FAFB. A similar approach has recently been described for MANC^22^. Note that proofreading across the FAFB-FlyWire dataset was reported in aggregate^2^ and that a first version of the neck connective annotations was released as part of the brainwide FlyWire annotations paper^3^.

### LM identification

DNs were identified by overlaying the EM-reconstructed DNs with images of Gal4 lines, mainly from the Namiki collection^5,34^ and Janelia’s Gal4 and Split-Gal4 collections^75–77^, or with the NeuronBridge tool^33,78^ for MANC DNs. To compare the reconstructions and LM images in the same space, the latter were segmented and transformed into MANC space as described previously^22^ or into FAFB space. The full list of DN types with the identifier for the LM image (slide_code) and for the type (VFB_ID) can be found in Supplementary File 4. A small list of ANs was also matched to LM in FAFB and MANC because these ANs were of particular interest for the circuit described in Fig. 3 (see Supplementary File 12). We did not match ANs to LM images systematically, owing to a lack of a catalogue describing these neurons (as is available for DNs^5^). SA neurons were divided into subclasses by comparing them to LM images of Janelia’s Gal4 and Split-Gal4 datasets using the NeuronBridge tool^33,78^ for MANC and then manually matching their axonal continuations into the brain to FAFB neuron reconstructions. Extended Data Fig. 2 shows the LM image of the genetic driver line that SA neurons were matched to, as well as the assigned long_tract and entry_nerve that were used to give SA neurons a subclass name, aiding their identification (see also Supplementary File 3).

The process of matching to LM images is not exhaustive (in part because LM images and genetic driver lines are not yet available for all neurons), and we ask the *Drosophila* community to contact the authors with missing identifications which can be reviewed and integrated into this resource.

### Matching of neurons across VNC datasets

FANC DNs and ANs were transformed into MANC space using the transform_fanc2manc function from the fancr R package (https://github.com/flyconnectome/fancr). This is a one-step thin plate spline transform based on 2,110 landmark pairs fitted to a complex transformation sequence mapping FANC to the JRCVNC2018F template^32^ and JRCVNC2018F to MANC^1^. A combination of NBLAST^74^, and connectivity analysis was used to identify candidate morphological matches. These were assessed manually and assigned MANC names if the match was of high confidence (confidences ranged from 1 to 5, high is >3; Supplementary Files 6, 7 and 11). In addition, all ANs that were not matched with high confidence were assigned hemilineage and soma location in FANC and were compared by two independent annotators within each hemilineage after a thorough review of the non-matching ANs to exclude reconstruction problems as a cause. ANs were first matched between FANC and MANC as individual neurons. We then reviewed these MANC–FANC matches to ensure that they respected the groups of neurons previously defined in MANC, thus providing an additional layer of validation. Cosine similarity as well as the identity of strong upstream and downstream synapses partners was used to help resolve ambiguous cases.

### Tract identification

VNC longitudinal tracts for MANC ANs, MANC SAs and FANC DNs were identified as previously described^22^. In brief, neurons were simplified to their longest neurite starting from the VNC entry point at the neck, and subsequently NBLAST clustered^74^. The clusters were manually assigned a tract by overlaying with tract meshes made for MANC^22^.

Analysis of AN tracts revealed that one cluster did not match any of the previously published DN tracts. This new tract was given the name AN-specific dorsal medial tract (ADM) in accordance with the tract naming of a previous report^79^.

### Neuropil identification

Primary brain neuropils were assigned in the FAFB dataset using the per-neuron neuropil counts of presynapses for ANs and postsynapses for DNs in the 783 FAFB version (available for download at https://codex.flywire.ai/api/download). A single brain neuropil was assigned if 80% of all synapses were within that neuropil, and two neuropils were assigned as a name (primaryneuropil_secondaryneuropil) if combined they reached the 80% threshold and each contained at least 5%. We assigned 367 DNs and 282 ANs as innervating multiple neuropils (‘multi’) as they collected input or gave significant output (more than 20%) to more than two neuropils, respectively.

Primary VNC neuropils were assigned to all DNs and ANs in the MANC and FANC datasets as previously performed for MANC DNs^22^. For MANC AN synapses, we used the neuPrint synapse ROI information of the manc:v1.2. For FANC AN and DN synapses, we retrieved the synapses allocated to AN and DN IDs from the synapse parquet file, retrievable through FANC CAVE and available from the FANC community upon request (provided by S. Gerhard).

A single neuropil abbreviation was given to a DN or AN if they innervated a VNC neuropil with more than 80% of their pre- or postsynapses. The two-letter abbreviations nt, wt, hl, it, lt, fl, ml, hl, mv, ov and ad correspond to NTct, WTct, HTct, IntTct, LTct, LegNpT1, LegNpT2, LegNpT3, mVAC, Ov and ANm, respectively. In addition, DNs and ANs that innervated a combination of upper tectulum (ut) or leg neuropils (xl) with more than 80% of their pre- or postsynapses were given those abbreviations accordingly. Any neuron that did not fall into one of those two categories was grouped as xn, standing for multiple neuropils. ANs that contained only a soma and soma tract in the VNC were excluded from this neuropil analysis and referred to as XA, as previously described^23^.

If the neuropil names were inconsistent within a group or pair of neurons, we calculated the mean of the pre- or postsynapses to determine the assignment.

### Information flow ranking

The information flow ranking previously reported^2^ for FlyWire was subsetted for DNs and averaged by DN type. The information flow analysis is based on an algorithm implemented in a previous study^40^ (https://github.com/navis-org/navis). A low rank indicates a more direct connection from sensory inputs to that DN type.

### Sexually dimorphic and sex-specific neurons

DNs previously described as sex-specific, such as the female-specific oviDNs and male-specific pIP10, were matched to the available light-level data and are referred to as sex-specific throughout the paper. Other DNs and ANs that we could not match between the VNC datasets (between female and male) and that could not be matched to light-level data, but were well reconstructed and had a left–right partner, were considered to be potentially female- or male-specific, and are also referred to in the text and figures as sex-specific.

DNs such as DNa08, that exist in both sexes but are known to be dimorphic in morphology, were matched to light-level data and are referred to as sexually dimorphic (sex. dimorphic in the figures). Other DNs and ANs that we could confidently match across the two VNC datasets but that were dimorphic in morphology are also referred to as sexually dimorphic (sex. dimorphic in the figures). The following neurons were not considered even though they show morphological differences: (1) neurons presumed to be neuropeptidergic, because large morphological differences in neuronal arbour are common even between the left and the right side of the same individual^23^; (2) ascending histaminergic neurons (AHNs), which have been shown to have a difference in morphology that is not related to the sex of the animal^65^; and (3) neurons that innervate the abdominal ganglion, because there are problems in the FANC dataset that make it impossible to distinguish between a difference in reconstruction state and potential dimorphism (noted as reconstruction issues in the Supplementary files). Differences in the number of ANs or DNs of a type were not considered as dimorphism in this paper, because they occurred in neurons that we consider populations and whose numbers differed across the two sides of one individual. A difference in number is noted in the Supplementary files as biological variation, a match that is not ascending or a general matching problem, if one side was not found (Supplementary Files 14 and 15).

### Synapse density plots

To calculate the synapse density of sexually dimorphic and sex-specific neurons in the VNC, we collected all synapses of the identified neurons in each dataset (FAFB: cleft score > 50 applied). We then tiled the space that their synapses occupy into roughly isotropic voxels of 5-µm size and counted the synapses in each voxel. Synapses were then colour-coded by density and plotted in three-dimensional space.

### FANC neuron types

All FANC neurons that can be matched to MANC neurons are referred to by their MANC name, with an additional ‘f’ denoting female, when presented in comparative graphs or connectivity plots. All FANC neurons identified are listed in Supplementary Files 6, 9, 10 and 13.

FANC ANs and DNs that were not previously identified in LM and that could not be matched between datasets were assigned new type names. For ANs and DNs, the type names were given in accordance with the previously established systematic type names (DN-target neuropil abbreviation number or AN-hemilineage abbreviation number). To distinguish from the previous type names in MANC, the numbering starts at 999 and goes down.

### Connectivity

For connectivity graphs, we used a threshold of weight ≥ 10 and per cent output > 0.5% for the initial retrieval of partners of the neurons of interest. In the following step we added all MNs, SNs or SAs that connected to those with a weight ≥ 5 to adjust for known reconstruction problems in these neurons and for the fact that sensory neurons tend to make fewer synapses with their partners individually and connect as a population of the same sensory origin (reflected by their type). Once all neurons of interest had been defined, we took an all-by-all connectivity adjacency matrix, in which all values were converted to input per cent to the receiving neuron, averaged by type (unless otherwise indicated). The graphs shown in the figures note the additional per cent thresholds that were chosen for the nodes plotted in each graph. The Pearson correlation coefficient was calculated in Fig. 5a,b for synapse numbers left and right in FANC and MANC with the implementations in the cor() function of the stats R package.

The FlyWire and FANC datasets were both acquired by serial section transmission EM with voxel sizes of approximately 4 × 4 × 40 nm; synapse detection was done using the ‘synful’ software package^80,81^. By contrast, the MANC dataset was imaged by focused ion beam–scanning EM, giving isotropic voxels of (8 nm)^3^, and synapse prediction was done using the methods of the hemibrain dataset^82^. There is a larger total number of identified synapses in the MANC^1^ dataset than in the FANC^4^ one (75 million versus 45 million). We also observe lower numbers of downstream partners across the DN population (1.245:1 MANC:FANC ratio), and specifically in the case of DNa02 (40,418 versus 23,836 synapses). We suspect that these differences are mostly of technical origin.

In addition, we see a difference in synapse numbers across the two sides of the FANC dataset, which might be a sample preparation issue or a biological variation, because it is also reflected in neuron morphology and across a wide range of right-side neurons compared with their partners on the left side of the same dataset.

### Neuroglancer resource

To help compare the neurons described in our work, we created a Neuroglancer environment^83^ that displays meshes for all three datasets in a common space. This environment can be opened in any modern web browser (we use Google Chrome) by following the short URL https://tinyurl.com/NeckConnective.

We opted to use the Janelia FlyEM male CNS dataset as a single anatomically consistent target space for display on the basis of resources from a previous study^72^. FAFB-FlyWire neurons were transformed into the space of the male CNS brain using rigid and non-rigid consecutive registrations^72,84^. Meshes for MANC neurons are those released previously^1^; we then use Neuroglancer to apply an affine registration ‘on the fly’ to place them within the space of the VNC of the male CNS volume. We applied non-rigid transformations (fancr::transform_fanc2manc function described above) to put FANC neurons into MANC space, and then used the same MANC-to-male-CNS affine registration within Neuroglancer to complete the transformation into male CNS space. Metadata annotations are provided for the three datasets using the format Type_Side_Class format. At present, only the optic lobe portion of the male CNS EM volume has been released, but having all the data transformed into male CNS space means that this Neuroglancer scene can be modified with minimal effort to display the full male dataset when it becomes available.

### Reporting summary

Further information on research design is available in the Nature Portfolio Reporting Summary linked to this article.

## Online content

Any methods, additional references, Nature Portfolio reporting summaries, source data, extended data, supplementary information, acknowledgements, peer review information; details of author contributions and competing interests; and statements of data and code availability are available at 10.1038/s41586-025-08925-z.

## Supplementary information

Supplementary Files 1–16 Supplementary Files 1–16 and guide.

Reporting Summary

## Data Availability

The datasets used in this work are described in previous studies^1–4,23^, which are cited at appropriate locations throughout our manuscript. The primary data from this work have been contributed to three dataset sources. Codex is available at https://codex.flywire.ai/; neuPrint is available at https://neuprint.janelia.org/; and the FANC dataset is available by joining the FANC community (instructions can be found at https://github.com/htem/FANC_auto_recon/wiki#collaborative-community). For ease of access, we also provide spatially integrated versions of the datasets, as well as access to the specific annotations in this paper. We provide a Neuroglancer scene, preconfigured for display and query of our annotations across all three datasets: https://tinyurl.com/NeckConnective. In this space, FANC neurons can be co-visualized with MANC neurons. We also provide a GitHub repository from which the annotations can be downloaded: https://github.com/flyconnectome/2023neckconnective. This GitHub repository includes neuron annotations; other metadata as provided in the supplementary files; a guide on how to use the Neuroglancer scenes; and example code and information on how to access the different datasets.

## Code availability

Analyses were performed using open-source packages with the R natverse^84^ infrastructure. The fafbseg, malevnc and fancr R packages have extensive functionality for working with FlyWire, MANC or FANC data, including querying annotations, fetching connectivity and working with the segmentation. As an entry point, we recommend the coconatfly R package, which has a uniform interface for analysis across datasets. The key software packages are navis (https://github.com/navis-org/navis) v.1.5.0, stats (https://www.r-project.org/) v.4.2.3, fafbseg-py (https://github.com/navis-org/fafbseg-py) v.3.0.5, flybrains (https://github.com/navis-org/navis-flybrains) v.0.2.9, skeletor (https://github.com/navis-org/skeletor) v.1.2.3, fafbseg (https://github.com/natverse/fafbseg) v.0.14.0, coconatfly (https://github.com/natverse/coconatfly) v.0.2.0, malevnc (https://github.com/natverse/malevnc) v.0.3.0 and fancr (https://github.com/flyconnectome/fancr) v.0.4.0. Example code specifically for the data used in this manuscript is provided in the accompanying GitHub repository (https://github.com/flyconnectome/2023neckconnective). This includes neuron annotations and other metadata collected in this manuscript.
