# Supplementary material for: Comparative connectomics of Drosophila descending and ascending neurons
Source: Nature. 2025 Apr 30;643(8070):158–72. doi: 10.1038/s41586-025-08925-z (PMC12222017; doi:10.1038/s41586-025-08925-z)
Supplement: Supplementary file 1 — Supplementary Files 1–16 and guide. [file 41586_2025_8925_MOESM1_ESM.zip › 2024-06-12178B-s1/2024-06-12178B-SIGuide.docx]

## Supplementary information (SI) guide

| Filename | Title | Description |
| --- | --- | --- |
| Supplemental_file1_FAFB_seed_plane.tsv | Supplementary File 1 | Tab-separated value (TSV) file containing the xyz coordinates, svid, root_id, side and class of profiles passing through the FAFB seed plane. |
| Supplemental_file2_FANC_seed_plane.tsv | Supplementary File 2 | Tab-separated value (TSV) file containing the xyz coordinates, svid, cell_id, root_id, side and class of profiles passing through the FANC seed plane. |
| Supplemental_file3_FAFB_SA_identification.tsv | Supplementary File 3 | Tab-separated value (TSV) file summarising the SA subclass identification in the FAFB dataset with the reference to light microscopy images taken from genetic driver lines. |
| Supplemental_file4_DN_identification.tsv | Supplementary File 4 | Tab-separated value (TSV) file containing the slide code of light microscopy images taken from genetic driver lines to identify DNs. |
| Supplemental_file5_FAFB_DNs.tsv | Supplementary File 5 | Tab-separated value (TSV) file containing the neuronal ids, types and annotations used in the manuscript for DNs of the FAFB dataset. |
| Supplemental_file6_FANC_DNs.tsv | Supplementary File 6 | Tab-separated value (TSV) file containing the neuronal ids, types and annotations used in the manuscript for DNs of the FANC dataset. |
| Supplemental_file7_MANC_DNs.tsv | Supplementary File 7 | Tab-separated value (TSV) file containing the neuronal ids, types and annotations used in the manuscript for DNs of the MANC dataset. |
| Supplemental_file8_FAFB_ANs_SAs.tsv | Supplementary File 8 | Tab-separated value (TSV) file containing the neuronal ids, types and annotations used in the manuscript for ANs and SAs of the FAFB dataset. |
| Supplemental_file9_FANC_ANs.tsv | Supplementary File 9 | Tab-separated value (TSV) file containing the neuronal ids, types and annotations used in the manuscript for ANs of the FANC dataset. Includes the matching to ANs of the MANC dataset. |
| Supplemental_file10_FANC_SAs.tsv | Supplementary File 10 | Tab-separated value (TSV) file containing the neuronal ids, types and annotations used in the manuscript for SAs of the FANC dataset. |
| Supplemental_file11_MANC_ANs.tsv | Supplementary File 11 | Tab-separated value (TSV) file containing the neuronal ids and annotations used in the manuscript for ANs of the MANC dataset. Includes the matching to ANs of the FANC dataset. |
| Supplemental_file12_AN_identification.tsv | Supplementary File 12 | Tab-separated value (TSV) file containing the slide code of light microscopy images taken from genetic driver lines to identify 3 AN types. |
| Supplemental_file13_other_MANC_FANC_matching.tsv | Supplementary File 13 | Tab-separated value (TSV) file containing the neuronal ids, types and annotations used in the manuscript that are not DNs, ANs or SAs. |
| Supplemental_file14_dimorphic_DNs.tsv | Supplementary File 14 | Tab-separated value (TSV) file containing the neuronal ids, types and annotations of dimorphic or sex-specific DNs from all three datasets. |
| Supplemental_file15_dimorphic_ANs.tsv | Supplementary File 15 | Tab-separated value (TSV) file containing the neuronal ids, types and annotations of dimorphic or sex-specific ANs from all three datasets. |
| Supplemental_file16_User_edits.xlsx | Supplementary File 16 | Microsoft Excel (XLSX) file containing four tables listing the number of edits to the neck connective neurons in FANC and FAFB summarised by lab or showing the edits made by the one person per lab that contributed the most. |
