# Supplementary material for: Comparative connectomics of Drosophila descending and ascending neurons
Source: Nature. 2025 Apr 30;643(8070):158–72. doi: 10.1038/s41586-025-08925-z (PMC12222017; doi:10.1038/s41586-025-08925-z)
Supplement: Supplementary file 2 — Reporting Summary [file 41586_2025_8925_MOESM2_ESM.pdf]

Corresponding author(s): Gregory Jefferis and Katharina Eichler

Last updated by author(s): Feb 14, 2024

Reporting Summary

Nature Portfolio wishes to improve the reproducibility of the work that we publish. This form provides structure for consistency and transparency in reporting. For further information on Nature Portfolio policies, see our Editorial Policies and the Editorial Policy Checklist.

Statistics

For all statistical analyses, confirm that the following items are present in the figure legend, table legend, main text, or Methods section.

n/a	Confirmed	
☒	☐	The exact sample size (*n*) for each experimental group/condition, given as a discrete number and unit of measurement
☒	☐	A statement on whether measurements were taken from distinct samples or whether the same sample was measured repeatedly
☒	☐	The statistical test(s) used AND whether they are one- or two-sided<br>*Only common tests should be described solely by name; describe more complex techniques in the Methods section.*
☒	☐	A description of all covariates tested
☒	☐	A description of any assumptions or corrections, such as tests of normality and adjustment for multiple comparisons
☒	☐	A full description of the statistical parameters including central tendency (e.g. means) or other basic estimates (e.g. regression coefficient) AND variation (e.g. standard deviation) or associated estimates of uncertainty (e.g. confidence intervals)
☒	☐	For null hypothesis testing, the test statistic (e.g. *F*, *t*, *r*) with confidence intervals, effect sizes, degrees of freedom and *P* value noted<br>*Give P values as exact values whenever suitable.*
☒	☐	For Bayesian analysis, information on the choice of priors and Markov chain Monte Carlo settings
☒	☐	For hierarchical and complex designs, identification of the appropriate level for tests and full reporting of outcomes
☐	☒	Estimates of effect sizes (e.g. Cohen's *d*, Pearson's *r*), indicating how they were calculated

Our web collection on statistics for biologists contains articles on many of the points above.

Software and code

Policy information about availability of computer code

Data collection	Does not apply.
Data analysis	Analyses were performed using open-source packages using the R natverse infrastructure. The fafbseg, malevnc and fancr R packages have extensive functionality dedicated to working with FlyWire, MANC or FANC data, including querying annotations, fetching connectivity and working with the segmentation. As an entry point we recommended the coconatfly R package which has a uniform interface for analysis across datasets<br>The key software packages are:<br>- navis: https://github.com/navis-org/navis v1.5.0<br>- stats: https://www.r-project.org/ v4.2.3<br>- fafbseg-py: https://github.com/navis-org/fafbseg-py v3.0.5<br>- flybrains: https://github.com/navis-org/navis-flybrains v0.2.9<br>- skeletor: https://github.com/navis-org/skeletor v1.2.3<br>- fafbseg: https://github.com/natverse/fafbseg v0.14.0<br>- coconatfly: https://github.com/natverse/coconatfly v0.2.0<br>- malevnc: https://github.com/natverse/malevnc v0.3.0<br>- fancr: https://github.com/flyconnectome/fancr v0.4.0<br><br>Example code specifically to the data used in this manuscript is provided in the accompanying GitHub repository (https://github.com/flyconnectome/2023neckconnective). This includes neuron annotations and other metadata collected in this manuscript.

For manuscripts utilizing custom algorithms or software that are central to the research but not yet described in published literature, software must be made available to editors and reviewers. We strongly encourage code deposition in a community repository (e.g. GitHub). See the Nature Portfolio guidelines for submitting code & software for further information.

Data

Policy information about availability of data

All manuscripts must include a data availability statement. This statement should provide the following information, where applicable:

- Accession codes, unique identifiers, or web links for publicly available datasets
- A description of any restrictions on data availability
- For clinical datasets or third party data, please ensure that the statement adheres to our policy

The datasets used in this work are described in Dorkenwald et al. 2024, Schlegel et al 2024, Takemura et al. 2024, Marin et al 2024, and Azevedo et al. 2024, and is cited at appropriate locations throughout our manuscript.

The primary data from this work have been contributed to the three dataset sources:
- Codex (https://codex.flywire.ai/)
- Neuprint (https://neuprint.janelia.org/)
- FANC dataset is available by joining the FANC community. Instructions on joining the FANC community can be found at https://github.com/htem/FANC_auto_recon/wiki#collaborative-community

For use of access we also provide spatially integrated versions of the datasets as well as access to the specific annotations in this paper. We provide a Neuroglancer scene, preconfigured for display and query of our annotations across all three datasets: https://tinyurl.com/NeckConnective. In this space FANC neurons can be co-visualised with MANC neurons. We also provide a GitHub repository from which the annotations can be downloaded: https://github.com/flyconnectome/2023neckconnective .
This github repository includes:
- neuron annotations + other metadata as provided in the supplementary files
- a guide on how to use the Neuroglancer scenes.
- example code and information on how to access the different datasets

Research involving human participants, their data, or biological material

Policy information about studies with human participants or human data. See also policy information about sex, gender (identity/presentation), and sexual orientation and race, ethnicity and racism.

Reporting on sex and gender	Does not apply.
Reporting on race, ethnicity, or other socially relevant groupings	Does not apply.
Population characteristics	Does not apply.
Recruitment	Does not apply.
Ethics oversight	Does not apply.

Note that full information on the approval of the study protocol must also be provided in the manuscript.

Field-specific reporting

Please select the one below that is the best fit for your research. If you are not sure, read the appropriate sections before making your selection.

☒ Life sciences ☐ Behavioural & social sciences ☐ Ecological, evolutionary & environmental sciences

For a reference copy of the document with all sections, see nature.com/documents/nr-reporting-summary-flat.pdf

Life sciences study design

All studies must disclose on these points even when the disclosure is negative.

Sample size	We analyse neuronal reconstruction of 3 independent electron microscopy datasets from 3 different animals.
Data exclusions	No neurons were excluded from overall annotation or analyses.
Replication	There are currently no further EM datasets available that could be used to replicate this work.
Randomization	With only 3 datasets at hand randomization is not possible.
Blinding	With only 3 datasets blinding is not possible.

Reporting for specific materials, systems and methods

We require information from authors about some types of materials, experimental systems and methods used in many studies. Here, indicate whether each material, system or method listed is relevant to your study. If you are not sure if a list item applies to your research, read the appropriate section before selecting a response.

Materials & experimental systems

n/a	Involved in the study
☒	☐ Antibodies
☒	☐ Eukaryotic cell lines
☒	☐ Palaeontology and archaeology
☒	☐ Animals and other organisms
☒	☐ Clinical data
☒	☐ Dual use research of concern
☒	☐ Plants

Methods

n/a	Involved in the study
☒	☐ ChIP-seq
☒	☐ Flow cytometry
☒	☐ MRI-based neuroimaging

Plants

Seed stocks	Does not apply.
Novel plant genotypes	Does not apply.
Authentication	Does not apply.
